# Violent shaking versus a bicycle trailer ride: experimental comparison of kinematics in infant injury scenarios

**DOI:** 10.1098/rsos.260279

**Published:** 2026-10-28

**Authors:** Kim Hutchinson, Puck Bieseman, Jenny Dankelman, Ajay Seth, Arjo J. Loeve

**Affiliations:** ^1^ BioMechanical Engineering, Delft University of Technology, Delft, Zuid-Holland, The Netherlands; ^2^ Co van Ledden Hulsebosch Centre for Forensic Science and Medicine, Amsterdam, Noord-Holland, The Netherlands

**Keywords:** child abuse, biomechanical model, inflicted head injury by shaking trauma, head kinematics, abusive head trauma, bicycle, child transport

## Abstract

Fatal traumatic head injury in infants is regularly inflicted by a perpetrator, with head injury from shaking trauma (IHI-ST) being a major type. In IHI-ST, perpetrators violently shake an infant back-and-forth, whipping its head back-and-forth. Courts often debate whether shaking caused injuries or whether an alternative accidental scenario raised by the defendant did, like a bumpy bicycle-trailer ride. Head and torso kinematics of an infant dummy (six-week-old) during bicycle-trailer rides across bumps with different heights and slopes were recorded at different speeds. The validity of this alternative scenario was investigated by comparing it with violent shaking kinematics. Trailer ride conditions substantially affected the magnitude of dummy accelerations, velocities and neck flexion. Even for the highest speeds and steepest/highest bumps, head motion was less rotational than during violent shaking. While sagittal peak head linear accelerations were of similar magnitude, head angular accelerations in the trailer were less than half those during violent shaking. The head's median instantaneous radius of rotation was around 12 times shorter during shaking, indicative of greater rotational motion. Because of differences in kinematics, differences in the exposure duration, and the lack of injury thresholds between both scenarios, determining the resulting differences in injury risks requires further research.

## 1. Introduction

A common form of inflicted head injury (IHI), often referred to as abusive head trauma (AHT), is inflicted head injury by shaking trauma (IHI-ST) [1–4]. IHI-ST is often imposed by grabbing the victim's torso and fiercely accelerating it back-and-forth in its sagittal plane, thereby whipping its head back-and-forth. Injuries typically associated with IHI-ST include encephalopathy, as well as subdural and retinal haemorrhages [5–7]. However, these injuries are not unique to shaking and each can, particularly in isolation, have other causes. Therefore, concluding that shaking is the cause in cases of IHI also considers witness statements and the exclusion of alternative causes. Unfortunately, eyewitnesses and confessions are often lacking. Moreover, exact injury mechanisms of IHI-ST remain incompletely understood. Therefore, violent shaking as a cause of IHI is often debated in courts, with various alternative (often accidental) scenarios being argued.

A type of alternative scenario that has been raised in court cases involves the infant being shaken through other means, such as rough cradling, running with the infant in your arms or in a stroller, or a bumpy ride in a towed bicycle trailer, with the bumpy trailer ride being the focus of the current study [8–11]. This form of child transportation is generally considered by manufacturers and cycle unions as a safe alternative to bike-mounted child seats, though use instructions generally prohibit use with children less than 24 months old [12–15]. By contrast, when raised as an alternative scenario in legal cases, the defence instead frames trailer rides as potentially unsafe. The infant's prolonged exposure to a bumpy trailer ride, rather than inflicted violent shaking, is then blamed as the cause of injury. This argument implies that head kinematics during a bumpy trailer ride can be as injurious as the head motion responsible for injuries associated with IHI-ST. The only reported injuries related to trailer rides seem to involve children older than one year and injuries unrelated to those arising from prolonged repetitive shaking of the infant itself, calling into question the plausibility of the trailer ride as a valid alternative scenario for shaking trauma. Whole-body vibration of child dummies in a bicycle trailer has been investigated [16], but full head and torso kinematic motion studies during a trailer ride, which would allow for the comparison with violent shaking kinematics, have not yet been published.

Since real infants cannot be used to experimentally study injury scenarios, models have been used to investigate IHI-ST [17–19]. Quantitative kinematic outcomes from shaking studies using models usually cannot directly be used for injury risk assessment in infants. First, the biofidelity of existing infant models is unknown at best. Second, no validated injury thresholds suitable for IHI-ST exist [20]. Fortunately, these models can be used to study qualitative effects of parameters and comparisons between shaking scenarios. For instance, Jones *et al.* [21] found that decreasing neck stiffness had a positive effect on head accelerations during violent shaking, using an infant rigid body model. In another example, Hutchinson *et al.* [22] investigated the effect of infant age on violent shaking kinematics. Glowinski & Głowińska [23] compared infant dummy head and torso accelerations during violent shaking with short falls using a wavelet analysis, allowing them to compare these scenarios in both the time and frequency domain. In summary, although dummy motion during shaking cannot directly predict injury in infants, it does provide a kinematic reference against which scenarios can be compared. The validity of such scenario comparisons depends on the use of kinematic parameters that relate to the injury mechanisms of interest.

The exact causal mechanisms of IHI-ST remain unconfirmed. A common hypothesis is that sagittal rotational motion causes injuries associated with IHI-ST. The cerebrospinal fluid (CSF) surrounding the brain dampens blunt impact to the head, protecting it from peak translational accelerations. Conversely, that same fluid may lubricate motion of the brain relative to the skull during rotation, possibly causing ruptured bridging veins and higher brain tissue deformation. Studies using finite element models (FEMs) of eyes also relate sagittal rotation to higher tensile stresses in the retina, compared with other loading directions [24]. This suggests rotational loads may be more likely to induce retinal haemorrhages, aligning well with IHI-ST victims often having extensive, multi-focal retinal haemorrhages [4,25]. One way of describing whether a motion of an object is predominantly rotational or translational is by using its instantaneous centre of rotation (ICOR). Any planar motion of a rigid object can at any instant be described entirely as a rotation around its ICOR. We call the distance from the ICOR to the object's centre of mass (CoM) the instantaneous radius of rotation (IROR). So, a motion described by a small IROR is predominantly rotational, while a large IROR involves a more translational motion. Consequently, if high peak angular accelerations with corresponding small IRORs occurred during a trailer ride, and if these are similar in magnitude to those in shaking, then trailer-induced head motion might pose a comparable head injury risk to shaking. Conversely, if peaks are lower and coincide with larger IRORs (predominantly translational motions), the trailer scenario is less likely to result in the loading that may cause IHI-ST.

To the best of our knowledge, no studies have yet been published on infant kinematics during a bike trailer ride, beyond vibration-related comfort studies. Therefore, the goal of the current study was to evaluate whether trailer-induced motion could plausibly result in head and torso kinematics similar to those in violent shaking, by measuring head and torso kinematics of an infant dummy during controlled bicycle trailer rides.

## 2. Methods

### 2.1. Experimental protocol

An infant dummy was seated in a trailer, towed by a bicycle at an approximately constant speed across custom-made bumps (figure 1A). The position, orientation, linear accelerations and angular velocities of the infant dummy's head and torso were recorded. Eleven conditions were tested, consisting of different combinations of bump types (§2.2.1) and trailer speeds. Trailer speeds were arbitrarily chosen as low (10 km h^–1^), mid (15 km h^–1^) and high (20 km h^–1^) speeds that were achievable with both a regular and electric bicycle. Collectively, the selected bump types and cycling speeds reflected realistic and worst-case user conditions. Details on trailer speed and bump type per scenario are provided in table 1. Bicycle riding was performed by the three researchers who set up the experiment (K.H., P.B. and A.J.L.), for three repeats per test condition at three speeds, producing a total of nine trials per condition and 99 trials in total. The trial order was randomized in blocks per bump type. For each bump type, different combinations of cyclists and speeds were randomized in MATLAB using the *randperm()* function.

**Figure 1.**
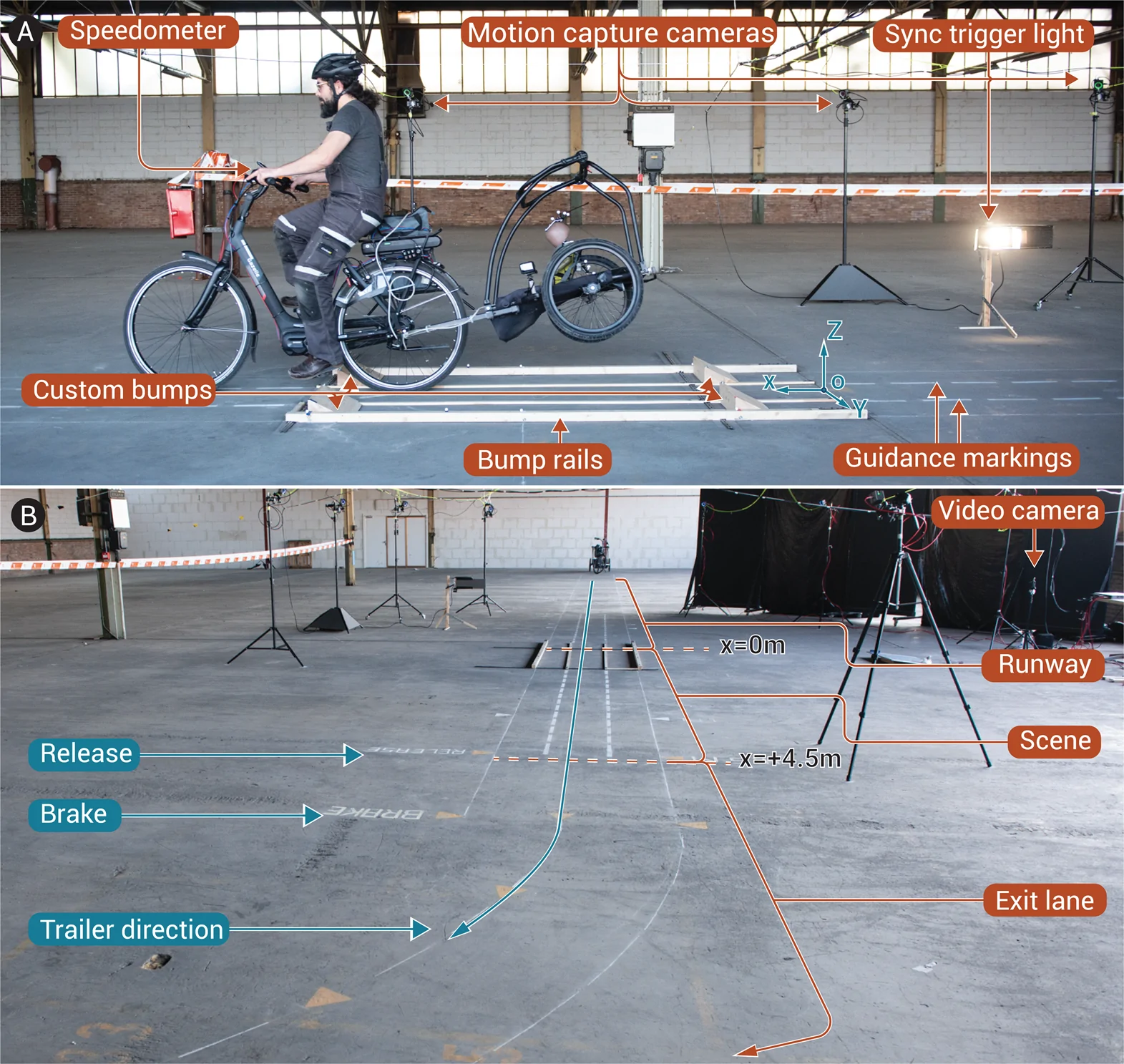
Overview of the experiment and set-up. (A) Impression of the experimental scenario. The cyclist was instructed to ride through the scene at a certain speed, which they monitored with the speedometer. The cyclist cycled between the bumps, which were fixed to the bump rails. The edges of the bumps were indicated by dashed lines on the floor before the track. Before the cyclist arrived at the scene, they passed the sync trigger light box. This light triggered an optical signal to the motion capture system and an electrical signal to the sensor processing software, allowing data synchronization. Three of the motion capture cameras and the global reference frame are also shown. (B) Overview of the experimental set-up, in which the different phases of each trial are indicated: the runway, scene (from *x* = 0 m to *x* = +4.5 m) and exit lane. The release, brake and trailer direction guidance markers for the cyclist are also indicated.

**Table 1.** Overview of the independent variables and the corresponding bump type (figure 2) per test condition.

test condition label	bump type label	speed (km h^–1^)	bump slope (°)	bump height (cm)
1	A	10	26	2.5
2	A	15	26	2.5
3	A	20	26	2.5
4	B	10	45	5
5	C	10	26	5
6	C	15	26	5
7	C	20	26	5
8	D	10	14	5
9	D	15	14	5
10	D	20	14	5
11	E	10	26	10

An additional set of explorative experiments was conducted for a subset of the main test conditions, except with an extra bump in the track, to explore if a resonance frequency would be reached that would exacerbate head accelerations. The data resulting from these additional tests were not included in the presented results but are included in the online open-access dataset. Details of these test conditions are included in appendix A in the supplementary material.

### 2.2. Experimental set-up and equipment

#### 2.2.1. Cycling track design

Five realistic and worst-case bump types were derived from field measurements of common irregular ground surfaces, such as curb ramps and sewer lids in Delft, the Netherlands (supplementary material, appendix B). These consisted of combinations of a 14°, 26° or 45° slope angle and a 25, 50 or 100 mm height (figure 2C). Bumps were fabricated out of pinewood (figure 2B,D) and screwed to custom-made tracks that were fixed to the ground (figure 2D). The bumps were designed so that only the trailer would hit them, and the bicycle would pass between them at the set target speed.

**Figure 2.**
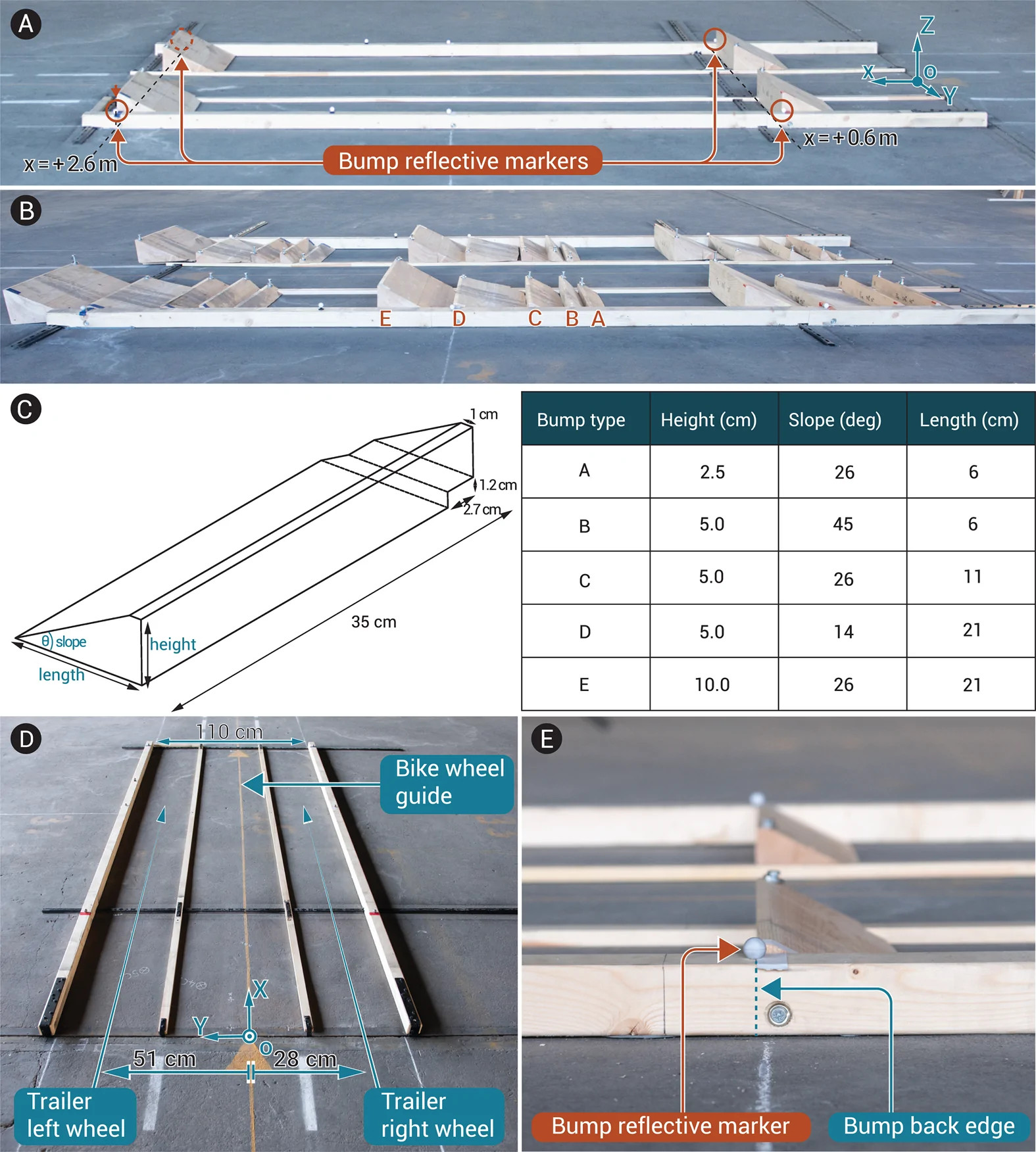
Overview of the bump set-up. (A) The x-positions of the back edges of the bumps and each corresponding reflective marker are shown, as well as the global reference frame. (B) Overview of the five types of bumps, with each corresponding type letter. (C) Dimensions of the bump profile per type. The schematic drawing depicts the right side of the bump of each type; the left side of the bump is mirrored. (D) Cyclist's view-point of the bump rails, which are 110 cm wide. The cyclist followed the yellow bicycle wheel guide, as in that position the left and right wheels of the trailer will crossthe middles of each bump. (E) Close-up of the side of a bump set, to show that the centre x-position of a bump reflective marker is located right against the back of the bump.

The parkour consisted of three consecutive regions (figure 1B: on the *runway*, the cyclist accelerated to the instructed speed, and aimed for the correct path, on the *scene* of 4.5 m long, the trailer crossed the bumps; and in the *exit lane*, the cyclist braked and exited the parkour. White guidance markings on the floor instructed the cyclist where to be at speed, aim, release and brake (see schematic top view in appendix C in the supplementary material). Dashed white markings indicated the edges of the bumps to help the cyclist aim between the sets of bumps (figure 1B), and a yellow line indicated where to aim the bicycle's front wheel so the trailer wheels would hit the middle of each bump (figure 2D).

#### 2.2.2. Bicycle and trailer

A Gazelle Orange C8 (Gazelle, Dieren, the Netherlands) electric bicycle was used to tow a modified 2010–2013 Honey Bee bicycle trailer (Burley Design, Eugene, Oregon, USA) (figure 3). The tyre pressure of the bicycle and trailer was intermittently checked (supplementary material, appendix D). The cyclist maintained the right speed using the speedometer mounted to the bicycle's handlebar (figure 3A).

**Figure 3.**
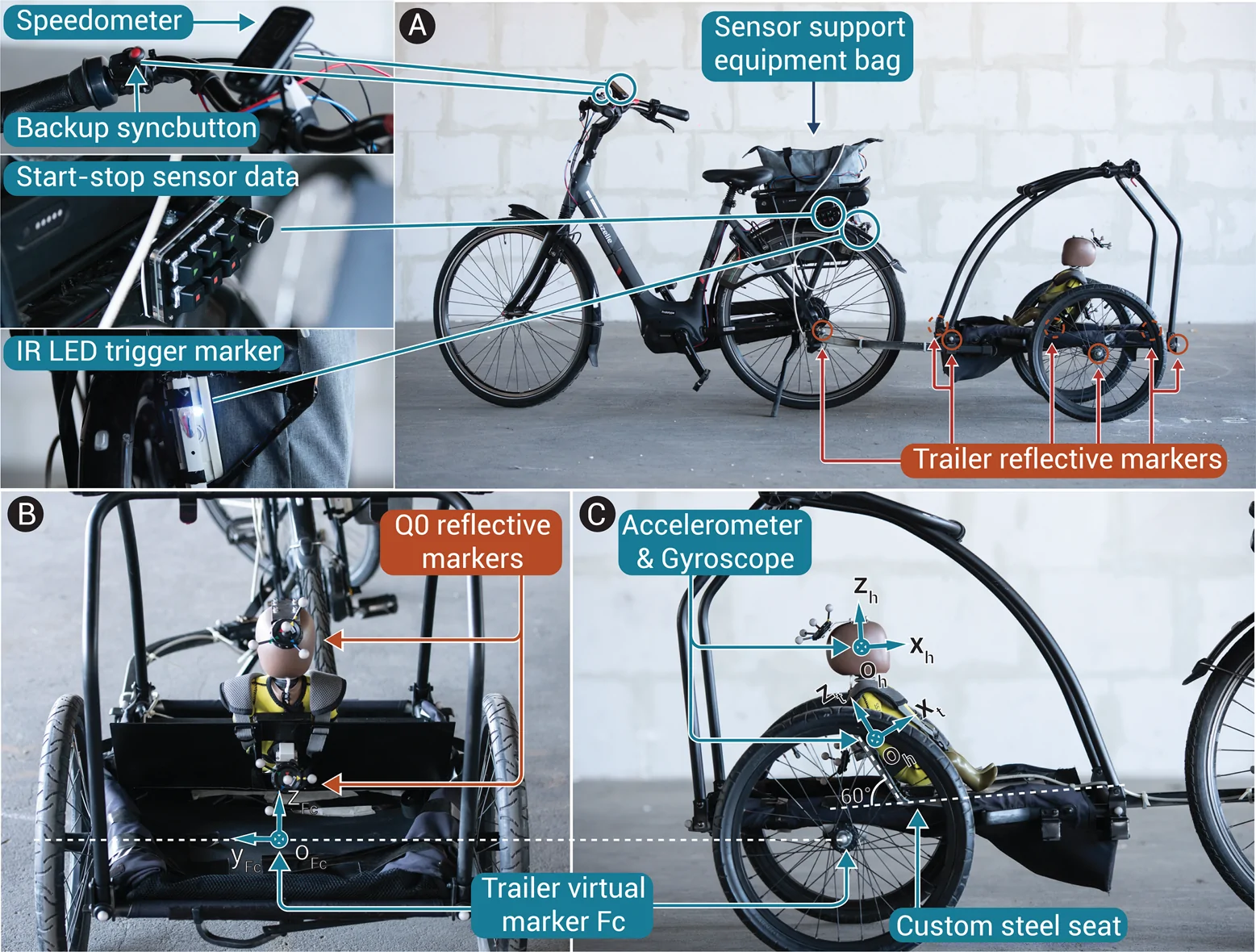
Overview of the bicycle trailer and Q0 dummy set-up. (A) Indication of the locations of the speedometer, the backup sync button and the start-stop buttons of the sensor data, which the cyclist pushed at the start and at the end of each trial, the location of the 850 nm IR LED trigger marker (not visible, but it is near the indicative light blue LED) and the locations of the seven reflective markers on the trailer. (B) The local reference frame of the trailer with its origin at virtual marker Fc is shown, positioned between the left wheel marker and the right wheel marker on the axle. The reflective markers of the Q0 are also indicated. (C) Depiction of the local reference frames of the head and torso and the respective reflective markers. The positioning of the custom steel seat, reclined at an angle of 60°, is also shown.

The trailer was modified in several ways. First, the fabric cover was removed to prevent marker occlusion. Second, the trailer's reflectors were removed and the frame was painted matte black to prevent ghost markers in the optical motion capture data. Finally, the trailer had a mesh headrest and seat, but this is not the case for all trailer models. To obtain a worst-case scenario, the headrest was removed and the seat replaced by a custom steel seat, reclined at 60°, matching the original seat's angle (figure 3C; supplementary material, appendix E). The dummy's torso was strapped to the seat.

#### 2.2.3. Instrumented infant dummy

The infant dummy used for the experiment was a Q0 crash test dummy (First Technology Safety Systems, Delft, the Netherlands). This dummy, representing a six-week-old infant, is the same one used in previous studies on violent shaking by Schiks *et al.* [26] and Hutchinson *et al.* [22]. It contains custom-built sensor brackets in the head CoM and in the torso. Each sensor bracket has one sagittal gyroscope (ADXRS649, measurement range 20 000° s^–1^, Analogue Devices, Inc., Norwood, Massachusetts, USA) and one tri-axial accelerometer (ADXL377, measurement range 200 g, Analogue Devices, Inc., Norwood, Massachusetts, USA). The maximum frequency response of both gyroscopes was adjusted from 88 to 884 Hz. Further details on the sensor system are described in Hutchinson *et al.* [22]. An overview of the connections to, from and inside the dummy and a description of the custom-built mobile power supply and its circuit are provided in appendix F in the supplementary material.

#### 2.2.4. Motion capture

The position and orientation of the trailer and the dummy's head and torso were tracked using eight OptiTrack *Prime^X^ 13* infrared motion capture cameras (NaturalPoint, Corvallis, Oregon, USA). Several ∅14 mm reflective markers were mounted to the rails to mark the end of each bump (figure 2A,E). Continuous calibration was enabled in the OptiTrack software, which used the positions of those markers to recalibrate if the cameras’ positions changed slightly during the experiment, e.g. through floor vibrations. A passive Traqr rigid body cluster (Qualisys, Göteborg, Sweden) containing four spherical ∅16 mm reflective markers was mounted to the head and torso, each, to track their positions (figure 3B,C). The trailer was tracked using seven ∅14 mm markers (figure 3A).

#### 2.2.5. Sensor and motion capture data synchronization system

The optical motion capture and sensor data were synchronized using a custom-built trigger system (see appendix F in the supplementary material for details) mounted to the back of the bicycle (figure 3A). The system consisted of a light-sensitive switch and an infrared LED. When the bicycle passed a lightbox at –2 m of the parkour (figure 1A), the switch triggered, which simultaneously sent a 5 V spike to the connected sensor data acquisition system and fired the LED. The motion capture system registered the LED as a marker, allowing the motion capture and sensor data to be synchronized.

#### 2.2.6. Video recordings

Each trial was filmed from the side of the track using a GoPro Hero 12 camera (figure 1B) for use as a reference and verification check during data analysis. For at least one trial per test condition, an additional GoPro Hero 8 camera was mounted to the trailer to either film the dummy or the direction the dummy was facing.

### 2.3. Data acquisition and processing

The sensor outputs of the gyroscopes and accelerometers in the Q0 dummy were recorded using a Data Acquisition card (DAQ) (NI USB-6211, National Instruments, Austin, Texas, USA) at a sample frequency of 5000 Hz, connected to a laptop running LabVIEW 2018. The motion capture data were recorded at 500 Hz, processed and exported using Motive:Body v. 3.1.4 (OptiTrack, NaturalPoint, Inc., Corvallis, Oregon, USA), the software accompanying the motion capture cameras. Connection schematics related to the sensor data collection can be found in appendix F in the supplementary material.

#### 2.3.1. Optical motion-capture data labelling

Markers were labelled in Motive:Body. While all measures were taken to prevent marker occlusions, they still occurred occasionally, resulting in gaps in marker trajectories. Gaps smaller than 25 frames for the head and 100 frames for the torso were generally filled using pattern-based interpolation (see appendix G in the supplementary material for details). All trajectories were then exported as .csv files for the data analysis.

#### 2.3.2. Adding virtual markers and their positions

The locations of the non-visible accelerometers inside the dummy and the axle midpoint of the trailer were added as *virtual markers* during processing. To relate these virtual markers to the visible markers, stationary calibration recordings were done with additional reference markers mounted to the dummy at known locations with respect to the virtual markers (supplementary material, appendix F, figure D6). The locations of the following four virtual markers were then computed and added to each frame during data processing:

— Ta: The position of the torso accelerometer (figure 3C),
— Ha: The position of the head accelerometer (figure 3C),
— Hv: The position of the head's vertex, and
— Fc: The position of the trailer's axle midpoint between its wheels (figure 3B).

### 2.4. Data filtering

Low-power high-frequency noise in the accelerometer data was removed using a 12th-order low-pass zero-phase-lag digital IIR Butterworth filter with a cut-off frequency at the accelerometer's frequency bandwidth of 500 Hz. A noise peak at 50 Hz was filtered out using a second-order digital infinite impulse response (IIR) Butterworth notch filter at a 50 Hz cut-off frequency with a bandwidth of 3 dB. Motion capture data trajectories were smoothed before ICOR analysis using the same 12th-order Butterworth filter, but with a cut-off frequency of 30 Hz.

### 2.5. Data analysis

Hutchinson *et al.* [22] found that accelerations in the sagittal plane were dominant during violent shaking. Therefore, the sensor data were *analysed* only in the local sagittal (x–z) planes of the head and torso. All data analysis was conducted using MATLAB (version R2024b, MathWorks, Natick, Massachusetts, USA)

#### 2.5.1. Sensor data

The following was computed using dummy sensor data of head (subscript *h*) and torso (subscript *t*):

— *ω_h_, ω_t_* (rad s^–1^): angular velocities recorded by single-axis gyroscopes in the local sagittal planes of head and torso.
— *α_h_, α_t_* (rad s^–2^): angular accelerations in the local sagittal planes, calculated by differentiating *ω_h_* and *ω_t_*, respectively, after applying a 0.02 s moving average filter window to reduce high-frequency noise.
— *a*_xh_*, a*_xt_*_,_ a*_yh_*, a*_yt_, *a*_zh_*, a*_zt_ (m s^–2^): linear accelerations in local x- (anterior/forward), y- (sinistral/left) and z- (superior/ upward) directions, obtained from the tri-axial accelerometers.
— *a*_xz_*_,h_, a*_xz_*_,t,_* (m s^–2^): magnitudes of the resultant linear accelerations in the local sagittal (x–z) planes.

#### 2.5.2. Neck flexion

The neck flexion angle θ (°) in the sagittal (ventral/dorsal) plane was computed using the motion capture data by calculating the angle between the z-axes of the torso and head in the local sagittal reference frame of the torso, with respect to the neck's neutral unperturbed pose.

#### 2.5.3. Instantaneous centre of rotation

The head CoM ICOR was calculated in the global x–z plane using the motion capture data for all data points between the moments the trailer marker Fc (figure 3B) was at *x* = 0 m and at *x* = 4.5 m . The ICOR was calculated as described by Hutchinson *et al.* [22], using interpolated, smoothed and up-sampled head, torso and trailer trajectory data matching the frame rate of the sensor data by calculating the:

— tangents to the trajectories of the head CoM virtual marker and the mean position of the head's marker cluster.
— Angle between the normals of these two tangent lines to check if they were collinear. If they were collinear, the ICOR could be either (a) close to collinear with these points on the head, causing the ICOR calculation to be inaccurate, or (b) very far from the head. To prevent numerical issues with collinearity, a minimum angle between the two normals of 5° was allowed. If the angle was smaller, point ‘b’ was replaced by head vertex position (Hv), eliminating collinearity.
— Intersection of the two normals, being the location of the ICOR. The absolute distance between the head's ICOR and CoM was defined as the IROR.

#### 2.5.4. Dummy and trailer shaking characteristics

The peak acceleration, maximum neck flexion and extension, and median IROR were calculated per trial, in the range from 0.05 m before the first bump started to Fc*_x_* = 4.5 mm (end of the scene). The peak outcomes in this range are referred to as the peak outcomes over the *whole trial* window.

At higher trailer speeds, the trailer often flew over the second bump and thus missed it, or just one bounce was recorded because the second landed when the Fc*_x_* position was beyond 4.5 m. To still be able to analyse possible effects of bump height, bump slope and trailer speed for trials missing the second bump, we defined a *first bounce* window: from 0.05 m before the first bump to the instance after the first landing when the neck flexion returned to neutral following a single forward and backward motion of the head. The same peak outcomes computed over the *whole trial* window were also computed over the *first bounce* window only (see appendix H in the supplementary material for details).

#### 2.5.5. Trailer velocity

To check whether the speed of the trailer during the trial actually matched the intended speed, and therefore whether trailer speed could indeed be tested using the intended groups, the x-position of the trailer marker Fc was differentiated to determine its velocity over time. The mean velocity across the whole trial was computed for each trial and its distribution was compared between groups.

### 2.6. Statistics

IBM SPSS Statistics 29 was used for statistical testing. Kruskal–Wallis tests were used to test for significant differences (*α* = 0.05) in the distributions of outcome measures between different bump slopes and heights and trailer speeds, using the following test conditions (table 1):

— **bump slope**: test conditions 8 (14°), 5 (26°) and 4 (45°) with bump height 50 mm and speed 10 km h^–1^,
— **bump height**: test conditions 1 (25 mm), 5 (50 mm) and 11 (100 mm) with bump slope 26° and speed 10 km h^–1^,
— **trailer speed**: test conditions 5 (10 km h^–1^), 6 (15 km h^–1^) and 7 (20 km h^–1^) with bump slope 26° and bump height 50 mm.

These tests were conducted for each of the following outcome measures:

— ***α_h_*** peak sagittal angular acceleration of the head (rad s^–2^),
— ***α_t_*** peak sagittal angular acceleration of the torso (rad s^–2^),
— ***ω_h_*** peak sagittal angular velocity of the head (rad s^–1^),
— ***ω_t_*** peak sagittal angular velocity of the torso (rad s^–1^),
— ***a*_xz_*_,h_****,* peak resultant sagittal linear acceleration of the head (m s^–2^),
— ***a*_xz_*_,t_*** peak resultant sagittal linear acceleration of the torso (m s^–2^),
— **θ_flex_** peak neck flexion (°),
— **θ_ext_** peak neck extension (°), and
— median IROR (m).

When significant differences were found, pairwise comparisons within each group were done using Dunn's post hoc tests (*α* = 0.05). Bonferroni correction was used to adjust *p*-values of the post hoc tests for multiple comparisons. Distributions of the outcome measures were tested for normality using Anderson–Darling tests. Most outcomes deviated significantly from a normal distribution (supplementary material, appendix I), which is why non-parametric tests were used.

### 2.7. Comparison with violent shaking

The kinematics of the infant dummy during the trailer ride were compared with previously published kinematics recorded during violent shaking of the same dummy by volunteers [22]. To this end, the distributions of the peak head sagittal angular velocity and acceleration, peak head resultant linear sagittal acceleration, peak neck flexion and extension and the head's IROR were plotted side by side.

## 3. Results

### 3.1 Overview

A total of 99 trials (excluding additional explorative trials) were conducted to obtain motion capture, accelerometer and gyroscope data of the infant dummy's head and torso during bicycle trailer rides with varying bump types and trailer speeds. Trial number 63 (part of the test set with bump C, speed 15 km h^–1^) had to be excluded because the visual trigger signal necessary for data synchronization was absent from the motion capture data. Outcome measures for both the whole trial and the first bounce are included in appendix I in the supplementary material. Significant effects of trailer speed, bump height and bump slope were found on almost all outcome measures. Pairwise post hoc comparisons indicated that only for bump height on torso angular acceleration no significant effect was found. Detailed results of all statistical tests are included in appendix I in the supplementary material. Analysis of the mean velocities per trial of the trailer was all within 2 km h^–1^ of the intended velocities, so no overlap in mean velocity between groups was found (supplementary material, appendix I).

### 3.2. Angular acceleration and velocity

Trailer speed had a strong positive effect on the peak angular acceleration and velocity of the head and torso in their local sagittal planes (figure 4c1,c2). Over the whole trial, the highest head angular accelerations and velocities were found in the group with bump type C and trailer speed 20 km h^–1^. Median acceleration values were 1933.5 (interquartile range (IQR) 1828.1–1993.2) and 760.3 (IQR 698.6–808.3) rad s^–2^ for the head and torso, respectively (figure 5A). Note that angular velocities and accelerations were only recorded in the dummy's sagittal plane.

**Figure 4.**
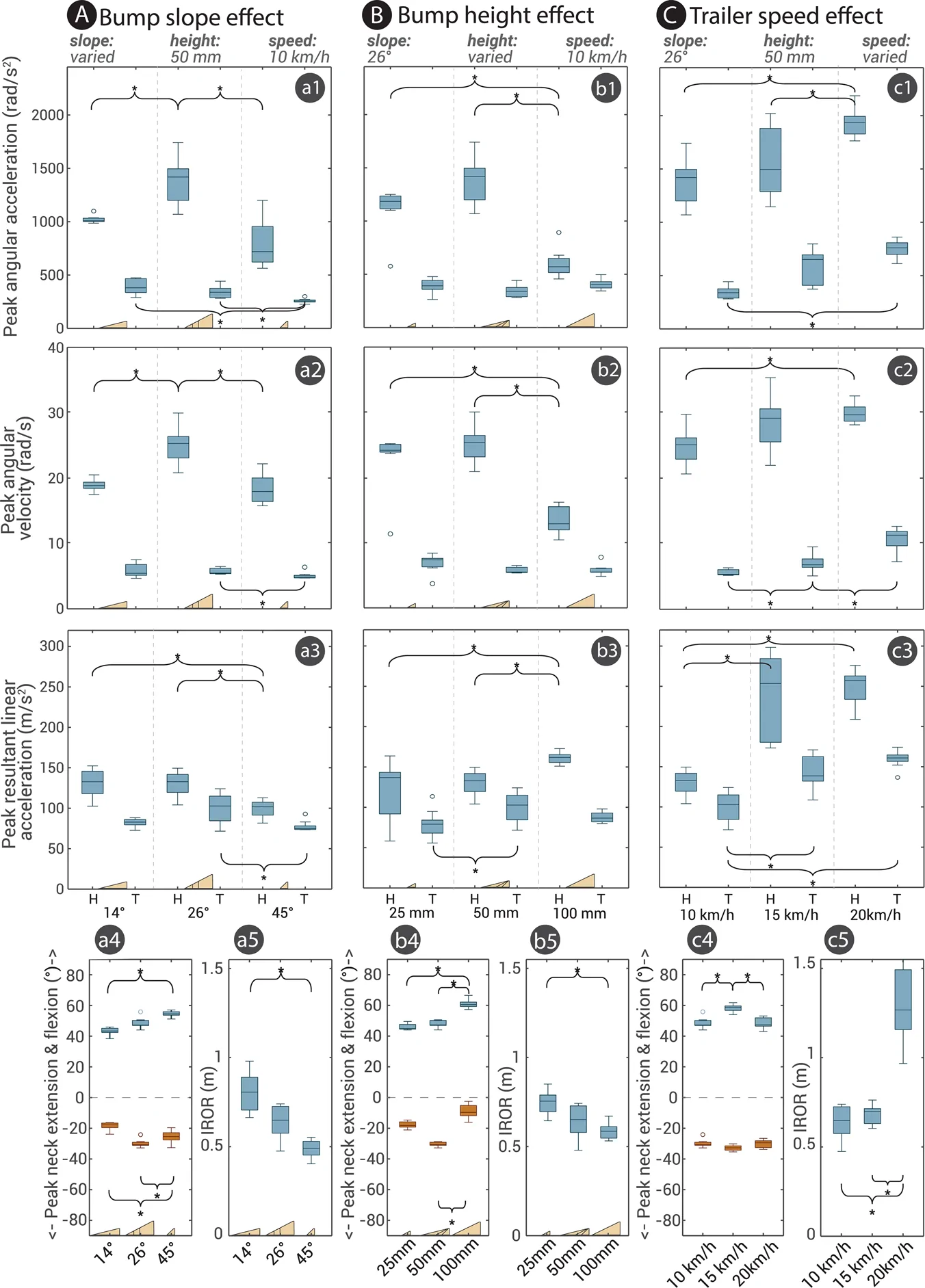
Distribution of the outcomes per testing group in rows: (1) peak angular acceleration, (2) peak angular velocity, (3) peak resultant linear acceleration, (4) peak neck flexion/extension and (5) median IROR during the first bounce of the trailer. Results are given per independent variable in the columns: (A) bump slope, (B) bump height and (C) trailer speed. Significant pairwise differences between slope, height or speed distributions are indicated with ‘*’. The profile of the corresponding bump types per group is illustrated on the horizontal axis.

**Figure 5.**
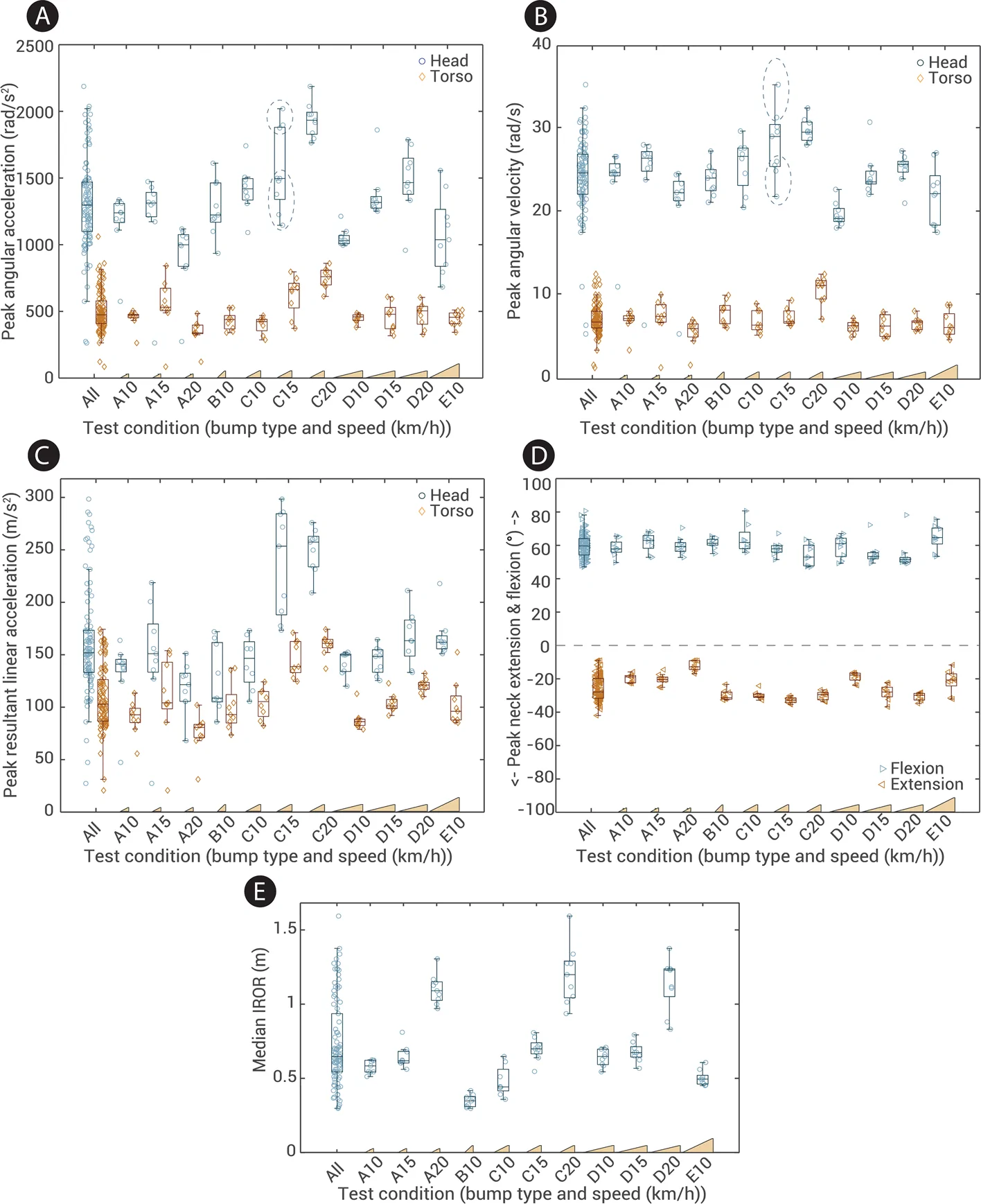
Scatterplots overlaid on boxplots of trial outcomes over the whole trial, for all trials and per test condition. Test conditions are indicated by bump type (A) (height 25 mm, slope 26°), (B) (height 50 mm, slope 45°), (C) (height 50 mm, slope 26°), (D) (height 50 mm, slope 14°) or (E) (height 100 mm, slope 26°) and a trailer speed (10, 15 or 20 km h^–1^). The profile of the corresponding bump is illustrated on the horizontal axis. The subplots show (A) peak angular acceleration of the head and torso in their local reference frames, (B) peak angular velocity of the head and torso in the local reference frames, (C) peak resultant linear acceleration in the local plane of head and torso, (D) peak neck flexion and extension and (E) median IROR of the head CoM. Outcome data clusters of head peak angular acceleration outcomes within group C15 are indicated with dashed ovals.

Bump slope and bump height were also both found to have a significant effect on head and torso angular acceleration and velocity, although the effect of slope was non-monotonic. The peak angular acceleration and velocity of the head were significantly lower during trials with a bump height of 100 mm than during 50 and 25 mm bumps (figure 4b1,b2). For a varying slope, the peak angular acceleration and velocity of the head were significantly higher at bumps of 26° than at 14° and 45° (figure 4a1,b1). The lowest peak head and torso angular accelerations were measured in the group with the steepest slope, B10 (height 50 mm, slope 45°), with medians 999.8 (IQR 837.4–1075.5) and 338.8 (IQR 333.0–397.3) rad s^–2^ for the head and torso, respectively.

### 3.3. Linear acceleration

The speed of the trailer with bump type C (height 50 mm, slope 26°), was found to have a significant positive effect on the peak resultant linear accelerations in the local reference frames of the head and torso (figure 4c3). For the trials with bump type C, the highest peak resultant linear accelerations of the head and torso over the whole trial were found during the highest trailer speed of 20 km h^–1^ (figure 5C). The median values of this group were 257.4 (IQR 233.8–262.9) and 160.9 (IQR 156.6–164.7) m s^–2^ for head and torso, respectively.

A significant effect of the bump slope and height was also found, although not as strong: the peak resultant linear acceleration slightly increased at higher bumps (figure 4b3) and decreased at steeper slopes (figure 4a3). Accordingly, the lowest peak linear accelerations over the whole trial were found in the group with the steepest slope (45°) at the lowest speed, group B1, with median values of 108.6 (IQR 105.1–161.6) and 80.6 (IQR 70.9–83.6) m s^–2^ for the head and torso, respectively.

### 3.4. Neck flexion

A positive significant effect of bump height on neck flexion was found with 100 mm high bumps resulting in significantly higher peak neck flexion than 25 and 50 mm bump heights (figure 4b4). The highest peak neck flexion was found in the group with bump type E (height 100 mm, slope 26°), with a median value of 68.6 (IQR 64.8–74.3°) m s^–2^. Neck flexion was also found to increase significantly at steeper bump slopes (figure 4a4). Notably, no significant difference was found between the peak neck flexion at 10 and at 20 km h^–1^, but the peak flexion at 15 km h^–1^ was significantly higher than at both 10 and 20 km h^–1^.

The highest absolute peak neck extensions were found with bump type C (height 50 mm, slope 26°) and speed 15 km h^–1^, with a median value of –32.8 (IQR –34.3 to –31.5)° m s^–2^. The absolute median peak neck extension was higher at a 26° slope than at a 14° and a 45° slope, and higher with a 50 mm height than with either a 25 or 100 mm height (figure 4a4,b4).

### 3.5. Instantaneous centre of rotation

A significant effect of trailer speed on median IROR was found (figure 4c5), with a significantly larger median IROR in the group with 20 km h^–1^ than with 10 and 15 km h^–1^. While trailer speed had a positive effect on the median IROR, both an increasing bump slope and height significantly decreased the median IROR (figure 4a5,b5). The lowest median IROR for the head was found for bump type B (height 50 mm, slope 45°), with a median value of 0.35 (IQR 0.31–0.38) m (figure 5E). The largest median IROR, indicating an overall more translational motion of the head, was found with bump type D (height 100 mm, slope 14°) and a trailer speed of 20 km h^–1^: 1.23 (IQR 1.05–1.24) m.

In figure 6, the x–z position of the ICOR with respect to the head CoM is depicted at the moment of the peak head angular acceleration during the first bounce (figure 6A,C) and over the whole trial (figure 6B,D). The axis range in figure 6C,D was set at ±0.5 m in the x- and z-directions as this is closer to the head CoM. Figure 6A,B with an axis range of ±2.5 m, show that in many trials the ICOR lies far away from the head CoM, beyond the 0.5 m range, indicating a more translational motion of the head at the moment of peak head angular acceleration in roughly half of the trials.

**Figure 6.**
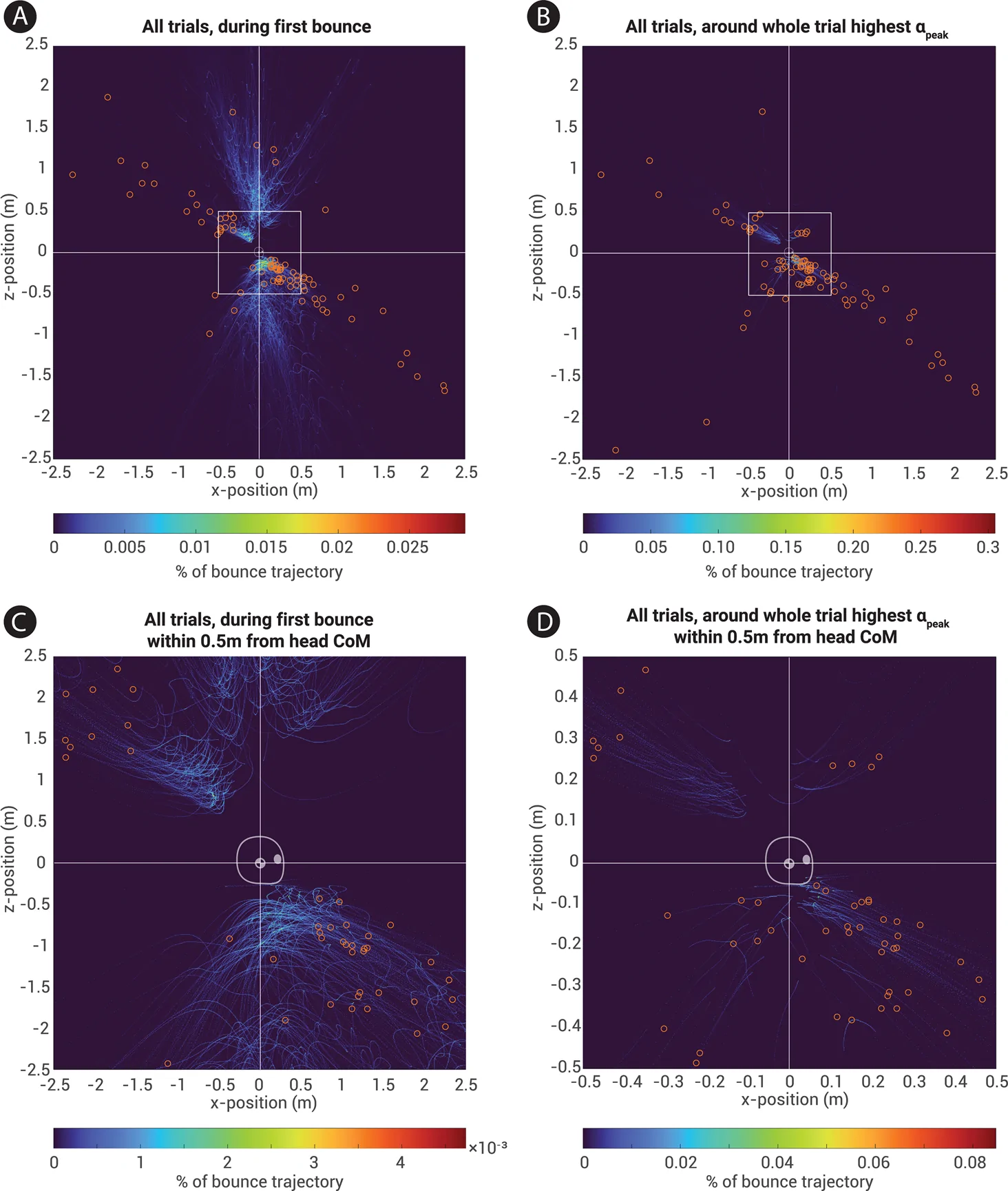
Heat maps displaying the percentage of time the ICOR was present at each x–z position during the trajectory (A,C) of the first bounce and (B,D) around the highest angular acceleration peak over the whole trial. This is indicated for all 98 included trials and is expressed with respect to the head local frame. Each orange circle of the scatter plot overlay indicates the position of the ICOR at the moment of the head peak angular acceleration of that trial. (A,B) show the ICOR range between –2.5 and +2.5 m (x- and z-direction) with the white square in A and B indicating the –0.5 to +0.5 m range that is enlarged in C and D, which is smaller and serves as a more relevant range for the comparison with violent shaking, as done previously by Hutchinson *et al.* [22]. The icons of the dummy head are to scale with respect to the figure axes.

### 3.6. Motion description

Figure 7 shows neck flexion, IROR, head linear xyz-acceleration (head's local frame), head angular velocity and acceleration, and trailer (Fc marker) z-position plotted against trailer x-position for two typical trials. The trailer was launched upon hitting the first bump. The dummy's neck, starting off in slight flexion, extended slightly as the trailer went into flight, accompanied by a sharp peak linear z-acceleration of the head. During flight, the neck returned to a slightly flexed position. Immediately after trailer landing (timestamps L1a and L1b in figure 7), the dummy's head slung backwards rapidly and then forwards, illustrated by the neck flexion and extension. During this backward and forward motion of the head, angular acceleration in the local sagittal reference plane of the head dropped and then rose rapidly. Upon landing, the absolute linear z- and x-accelerations of the head both peaked. In general, linear and angular head acceleration peaks occurred upon both traversing the bump and landing with the timing of the maximum peak magnitudes varying greatly between trials. In some trials, trailer roll and lateral head motion were observed (supplementary material, appendix I), in which cases increased local-frame head and torso y-accelerations were observed. In the few trials where this was most prominent, peak out-of-plane linear head accelerations were around half the magnitude of the peak resultant sagittal accelerations.

**Figure 7.**
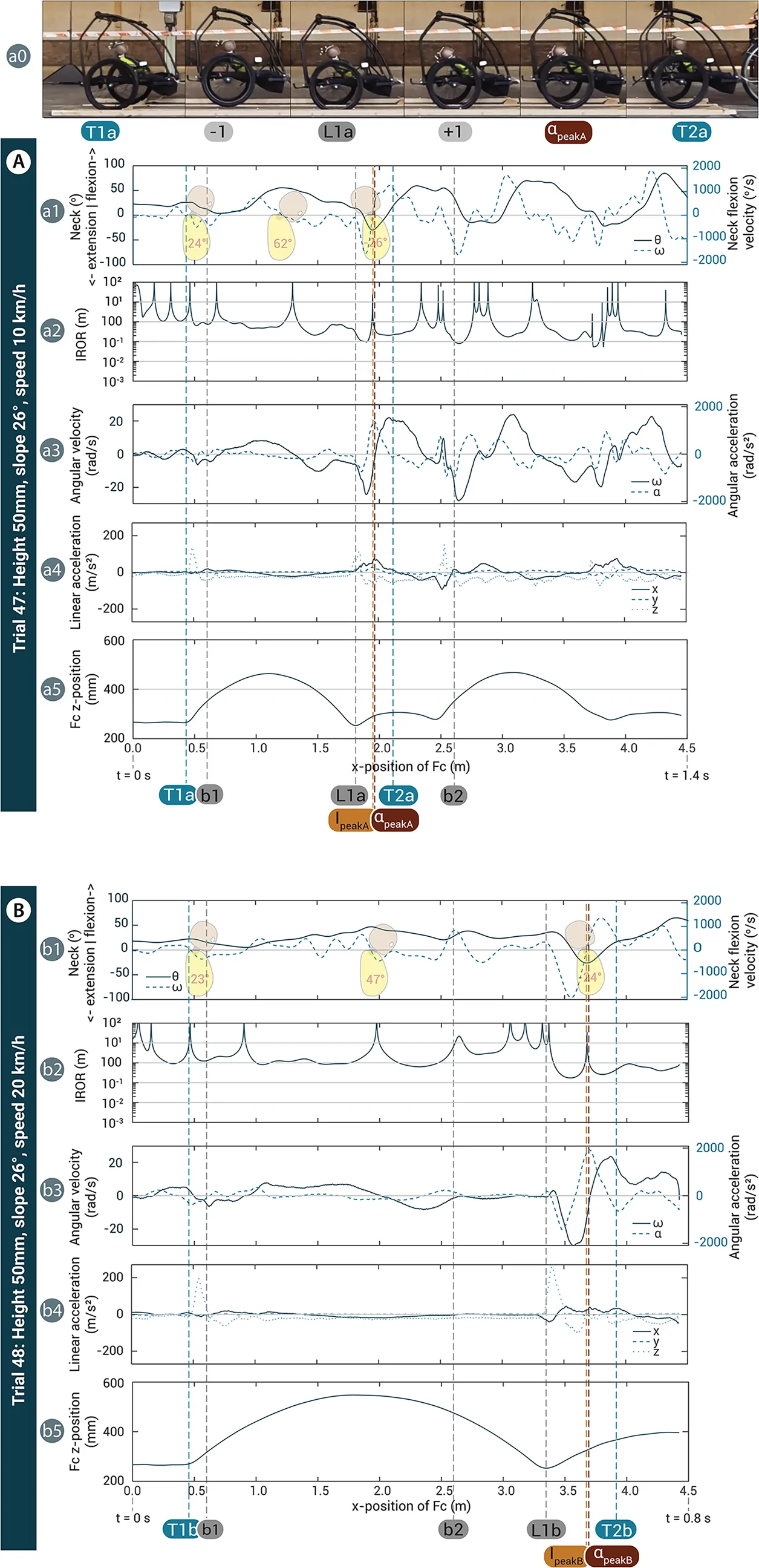
Two typical examples of trials with the same bump type (C: height 50 mm and slope 26°) but different trailer speeds (10 and 20 km h^–1^ for subfigures A and B, respectively). For both trials, the neck flexion and neck angular velocity (a1,b1), the IROR with respect to the local reference frame of the head (a2,b2), head angular velocity and acceleration (a3,b3), head linear acceleration (a4,b4) and the z-trajectory of marker Fc (a5,b5) were plotted over the x-position of trailer marker Fc. Bump locations are indicated by the triangle-shaped cross sections starting at *x* = 0.5 and 2.5 m. Vertical dashed lines indicate moments of interest. (b1,b2) The moment Fc passes bumps 1 and 2, respectively. (T1a/b): when the first bounce starts, based on the x-position 5 cm before bump 1. (T2a/b): the end of the first bounce, defined as the first time that the neck flexion is zero again after the first forward motion of the head after landing. (L1a/b): the first landing after the first bump. *I*_peakA/B_: the moment when the IROR peaks for the first time after L1a/b, right before the peak angular acceleration of that trial. *α*_peakA/B_: the peak angular acceleration after L1a/b where the neck is extended maximally. A0 displays side-view GoPro video screenshots from trial 47, each labelled with its approximate timestamp. The –1 and +1 labels indicate video frames one frame before and after the L1a timestamp, respectively.

**Figure 8.**
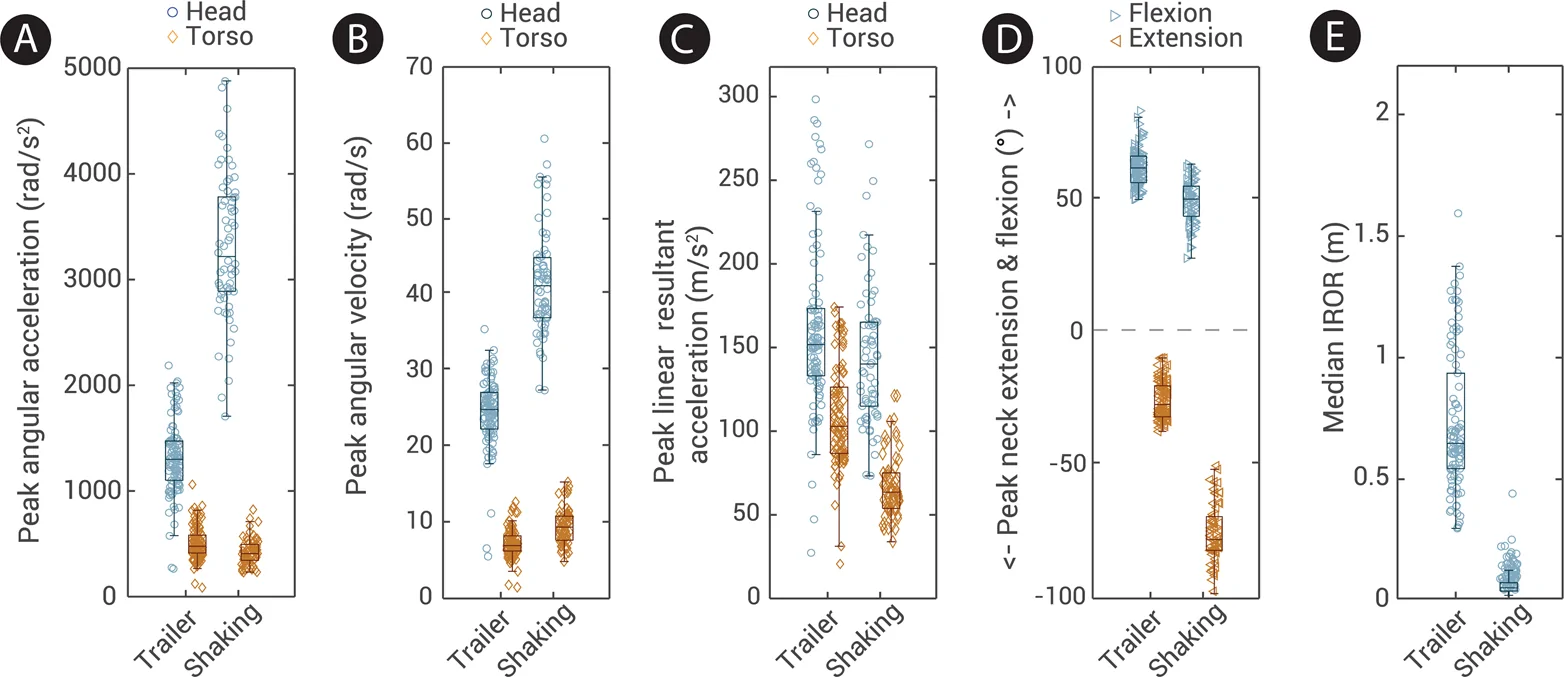
Scatterplots overlaid on boxplots of all bicycle trailer test conditions, next to data from violent shaking of the same dummy from Hutchinson *et al.* [22]. The subplots show (A) peak angular acceleration of the head and torso in their local reference frames, (B) peak angular velocity of the head and torso in their local reference frames, (C) peak resultant linear acceleration of the head and torso in their local reference frames, (D) peak neck flexion and extension and (E) median IROR of the head CoM.

### 3.7. Comparison with violent shaking

The distribution of peak head sagittal angular velocity and acceleration, peak head resultant linear sagittal acceleration, peak neck flexion and extension and the head's IROR were plotted side by side for the trailer ride and violent shaking in figure 8. There are particularly evident differences in angular velocities and accelerations of the head between the scenarios. The median peak angular acceleration of 3218.4 rad s^–2^, with a maximum of 4880.4 rad s^–2^, recorded during violent shaking was over twice the respective values found across all trailer rides in the current study (1302.6 and 2168.4 rad s^–2^). The peak angular velocity of the head was also higher during the shaking (median 41.2 rad s^–1^, maximum 60.5 rad s^–1^) than during trailer rides (median 24.9 rad s^–1^, maximum 35.5 rad s^–1^). This difference is again reflected in the IROR, with a median value of 0.054 m during shaking, 12 times smaller than during the trailer ride across all conditions. There is also a notable difference in peak neck extension between the scenarios, which was much larger during shaking than during the trailer ride (–79.5° versus –26.6°).

Distributions of peak angular accelerations and velocities of the torso do not appear to differ substantially between the shaking and trailer scenarios, either in medians or maximum and minimum values. The median and maximum magnitude of the peak sagittal linear acceleration of the torso are somewhat higher in the trailer scenario than during shaking, with medians of 102.9 versus 63.5 m s^–2^, respectively. There is no substantial difference in the distribution of the magnitude of the sagittal peak head linear accelerations between the scenarios, though there were some differences in the direction of sagittal linear accelerations. In the trailer scenario, peak linear z-accelerations of the head and mostly the torso were generally positive and of similar or higher magnitude than the x-accelerations, whereas in shaking they were generally negative and of lower magnitude than the x-accelerations.

## 4. Discussion

### 4.1. Overview

The head and torso kinematics of an infant dummy in a bicycle trailer, towed by an electric bicycle, were measured and characterized under controlled worst-case trailer conditions, to be compared with published kinematics of violent shaking. The trailer was towed over five custom bump types representative of urban street surfaces, with speed varied on three of the types. We provide a complete dataset of head and torso kinematics during a bicycle trailer scenario, enabling the first direct comparison with shaking kinematics with the same dummy and instrumentation.

### 4.2. Effect of trailer ride conditions

The conditions of the trailer ride substantially affected both the magnitude of the dummy's head accelerations and the nature of the motion. Overall, the highest peak linear and angular accelerations of the head occurred at the highest trailer speeds. The highest trailer speeds also resulted in the largest median IRORs, meaning the higher trailer speeds resulted in head motion that was less rotational in nature. Steeper and higher bumps resulted in smaller IRORs and thus a more rotational head motion. Interestingly, steeper slopes reduced the peak resultant linear acceleration of the head, whereas bump height increased it.

A monotonic increase or decrease was expected in the magnitude of peak sagittal head angular acceleration and neck flexion/extension with increasing bump slope and height (figure 4a1,b1). Instead, those peaks were higher at 26° than at 14° and 45°, at the same speed. The peaks were also higher for 50 than 100 mm bumps. In trials with the steepest and trials with the highest bumps, roll motion of the trailer was observed. This was probably caused by unequal vertical wheel forces resulting from the trailer's asymmetrical build (mounted on the left of the bicycle's rear axle only). The roll-motion induced sideways head motion, probably producing angular accelerations outside its sagittal plane. Although no angular accelerations outside the sagittal plane were recorded, head and torso absolute linear y-accelerations were indeed higher in trials with trailer roll (see supplementary material, appendix I, figure S4) than in trials without. As a result, resultant head angular accelerations for these trials in particular were probably actually higher than suggested by the sagittal values alone. In real-life situations such as riding up a curb diagonally or hitting a pothole with one wheel only, these out-of-plane motions are probably more prevalent still. Though this study's aim was comparison with violent shaking, where head motion occurs primarily sagittally, future research should ideally include axial and transverse angular velocities to enable comparison with other scenarios.

As expected, the head's peak resultant linear accelerations increased for higher bumps and trailer speeds (figure 4b3,c3). By contrast, steeper bumps reduced those same accelerations (figure 4a3). This may be owing to steeper bumps inducing more local tyre deformation, absorbing more impulse without transferring it to the trailer, reducing dummy accelerations (see video stills in appendix I in the supplementary material). Another explanation could be the wheels bouncing up off the ramp instead of rolling across the full ramp before flight with steeper bumps. Another unexpected result was found in the group with speed 15 km h^–1^ and bump C (height 50 mm, slope 26°), where peak head angular and resultant linear acceleration data seemingly separated into two subsets: trials with much higher and trials with much lower peak linear and angular head accelerations (figure 5A,C: test condition C15). In the subset with higher accelerations, we found the trailer landed directly on the second bump's face, making it bounce higher on that second bump. As such, landing on the face of the second bump probably explains the increased head peak linear acceleration in these trials. These outcomes emphasize the need to match conditions as precisely as possible when reconstructing a real case scenario, and more importantly, to reconstruct ranges of conditions for parameters that are not precisely known.

### 4.3. Comparison to violent shaking and safety implications

The ultimate goal of the current study was to evaluate whether a trailer ride should be considered a potentially valid alternative scenario to explain injuries typically associated with IHI-ST, by assessing whether it could plausibly induce head and torso kinematics similar to those in violent shaking. In terms of torso motion, the main difference between the shaking and trailer scenario is in the peak linear accelerations, which were somewhat higher in the trailer. This difference in peak acceleration is probably owing to the fairly hard impact contact between the trailer and the ground, whereas in shaking, the torso was not subjected to impact with any surfaces. In terms of head motion, the primary difference is the much higher peak angular velocities and accelerations in the shaking scenario. The extent of this difference means that only the harshest bumps produced results that overlapped only with the very mildest shaking results. Moreover, while in the shaking scenario the high peak angular head accelerations were accompanied by small IRORs, this was not the case in the trailer scenario. Finally, peak neck extensions were much higher during shaking than during the trailer ride. These differences in head motion can be explained by differences in the nature of the torso input motion. Violent shaking involves a constant repetition of shaking cycles, where the head follows the torso in a back-and-forth motion in its sagittal plane. By contrast, the torso motion in the trailer does not undergo a periodic back-and-forth motion, with the torso instead continuously having a positive forward velocity. Overall, the differences in outcome measures (figure 8) indicate that the head motion during the trailer ride was much less rotational in nature than in shaking. This is of particular interest since literature widely suggests the rotational head motion during shaking may be key to the injury mechanisms in IHI-ST [24,25,27–33], increasing both the risks of ruptured bridging veins and retinal haemorrhages.

A typical approach in estimating the risk of head injury using measured bulk head kinematics is to use injury thresholds. These thresholds should be used with caution and under specific conditions only, for several reasons [20]. First, they usually consider only whether injury would result from a single peak acceleration, like from impact. Peak accelerations below these thresholds, but occurring over a longer duration or repeatedly, may still cause injury, depending on the magnitude and exposure duration. In the case of a bicycle trailer ride, exposure involves a duration of minutes to hours. Second, these thresholds are almost always determined in animals, some in adult humans, and then sometimes scaled to fit humans, adults or infants [20]. As an example, Lintern *et al.* [34] suggested a subdural haemorrhage threshold for a combined angular acceleration and velocity of 15 777 rad s^–2^ and 57.6 rad s^–1^, respectively, for 4.5-month-old infants, based on measurements in non-human primates that were scaled to a human infant based on brain mass and age-dependent tissue properties. This threshold was not exceeded during the trailer ride in the current study, but this cannot necessarily be interpreted to mean that no injury would occur. It is hypothesized that for IHI-ST and similar injuries we should identify injury thresholds similar to those for hearing damage, which use both sound intensity and exposure duration. Finally, even if suitable thresholds were available, the biofidelity of existing infant dummies is unknown, at best. For example, the necks of most commercial infant dummies (including Q0) are often considered too stiff. Therefore, the magnitude of their head accelerations is not necessarily representative of those in a real infant, complicating the use of injury thresholds. The current study assumes the too-stiff neck did not majorly affect the comparison of kinematics, since it affects both scenarios. Nevertheless, it may be that it did not affect both scenarios equally, possibly affecting the magnitude of the reported differences between the scenarios.

To provide a more intuitive understanding of the order of magnitude of the head accelerations recorded during the trailer ride, we can compare them with previously reported kinematics during different everyday scenarios. For example, it has been estimated that rollercoaster rides induce peak sagittal angular accelerations of 139 to 502 rad s^–2^ in adults [35] versus the median of 1933.5 rad s^–2^ across all recorded bike trailer trials. Another study [36] estimated that peak linear head acceleration of an adult plopping backwards into a chair is around 100 m s^–2^, while across all trailer trials it was 257.4 m s^–2^. So, both the linear and angular head accelerations of the dummy during the trailer ride are generally higher than those estimated for adults during these two typical everyday activities. However, as mentioned, a comparison based only on peak values does not suffice for a proper assessment of injury risk. This, along with the general lack of valid injury thresholds for infants and particularly cyclic loading, means it remains difficult to translate results such as the current to hard injury thresholds. However, it still serves as a strong holistic comparison of the severity of different scenarios.

Test conditions during the current study were deliberately harsh; head motion during an average trailer ride is probably less severe. The vertical impacts of the trailer during landing were not attenuated by the custom-made rigid seat used to ensure a worst-case scenario. The original mesh seat would reduce peak linear accelerations, and the original seat's headrest probably would minimize neck extension, thereby strongly reducing sagittal head angular accelerations. This study highlights key kinematic differences between the shaking and trailer scenarios, but without further research we cannot conclude if an infant is less likely to sustain head injuries from a trailer ride than from violent shaking, for several reasons. First, although head angular accelerations and velocities during the trailer ride were much lower than during violent shaking, they were not negligibly small, and no suitable thresholds for infants are available that could indicate whether this angular acceleration magnitude may already be damaging with sufficient energy transfer. Second, the exposure duration to peak accelerations can differ substantially between a trailer ride (e.g. minutes up to hours on a continuous bumpy road) and violent shaking (a few to tens of seconds, possibly in repeated periods). Although measured peak head angular accelerations were much lower than during violent shaking, it is unknown whether a much longer exposure to these lower accelerations might still be harmful. Third, the simulated terrain may not be fully representative of terrain typically traversed with a trailer. Finally, the same points regarding exposure duration and lack of suitable thresholds apply to head peak resultant linear accelerations—depending on exposure duration and direction, we cannot yet exclude that they may still be injurious.

### 4.4. Key points

— The dummy's head peak angular acceleration and velocity during the recorded trailer rides were much lower than during violent shaking, while the linear acceleration peaks were of similar magnitude.
— The head's median IROR was much larger during trailer rides than during shaking, meaning the head motion was much less rotational in nature than during shaking.
— This study provides a basis for comparison with other scenarios simulated using the same dummy, though its results should not be used directly as representative of real infants as the dummy's biofidelity is limited.
— Key kinematic differences between the scenarios were identified, but further research is needed before the plausibility of the trailer ride as an alternative explanation for injuries typically associated with violent shaking can be either accepted or rejected, because:
  (i) while peak angular head accelerations were lower than in shaking, they were not necessarily negligibly small, and injury thresholds remain unknown;
  (ii) the exposure time to both peak linear and angular accelerations can be longer during a trailer ride than during violent shaking. Together with fierceness, the duration, number of repetitions and intervals between repetitions are expected to affect injury risk, but suitable thresholds do not exist yet.

## Supplementary Material



## Data Availability

The dataset generated during this project has been made available as an open access dataset in the 4TU Centre for Research Data Repository [37]. For questions about the dataset, contact the corresponding authors. Supplementary material is available online [38].

## References

[1] Parks SE , Kegler SR, Annest JL, Mercy JA. 2012 Characteristics of fatal abusive head trauma among children in the USA: 2003-2007 an application of the CDC operational case definition to national vital statistics data. Inj. Prev. 18, 193–199. (doi:10.1136/injuryprev-2011-040128) 22015935

[2] Nuño M , Ugiliweneza B, Zepeda V, Anderson JE, Coulter K, Magana JN, Drazin D, Boakye M. 2018 Long-term impact of abusive head trauma in young children. Child Abuse Negl. 85, 39–46. (doi:10.1016/j.chiabu.2018.08.011) 30144952

[3] Lind K , Toure H, Brugel D, Meyer P, Laurent-Vannier A, Chevignard M. 2016 Extended follow-up of neurological, cognitive, behavioral and academic outcomes after severe abusive head trauma. Child Abuse Negl. 51, 358–367. (doi:10.1016/j.chiabu.2015.08.001) 26299396

[4] Choudhary AK *et al.* 2018 Consensus statement on abusive head trauma in infants and young children. Pediatr. Radiol. 48, 1048–1065. (doi:10.1007/s00247-018-4149-1) 29796797

[5] Narang SK *et al.* 2025 Abusive head trauma in infants and children: technical report. Pediatrics 155, e2024070457. (doi:10.1542/peds.2024-070457) 39992695

[6] Ellingson KD , Leventhal JM, Weiss HB. 2008 Using hospital discharge data to track inflicted traumatic brain injury. Am. J. Prev. Med. 34, S157–S162. (doi:10.1016/j.amepre.2007.12.021) 18374268

[7] Christian CW , Block R, Committee on Child Abuse and Neglect, American Academy of Pediatrics. 2009 Abusive head trauma in infants and children. Pediatrics 123, 1409–1411. (doi:10.1542/peds.2009-0408) 19403508

[8] Arnhem-Leeuwarden G . ECLI:NL:GHARL:2018:5478. https://uitspraken.rechtspraak.nl/details?id=ECLI:NL:GHARL:2018:5478.

[9] Midden-Nederland R . ECLI:NL:RBMNE:2023:3450. https://uitspraken.rechtspraak.nl/details?id=ECLI:NL:RBMNE:2023:3450.

[10] Midden-Nederland R . ECLI:NL:RBMNE:2022:4730. https://uitspraken.rechtspraak.nl/details?id=ECLI:NL:RBMNE:2022:4730.

[11] Midden-Nederland R . ECLI:NL:RBMNE:2025:2043. https://uitspraken.rechtspraak.nl/details?id=ECLI:NL:RBMNE:2025:2043.

[12] Draitser M , Southern California Bicycle Attorneys. 2023 Are trailers safer than bike-mounted seats for your children? https://www.bikelawla.com/blog/2023/07/are-trailers-safer-than-bike-mounted-seats-for-your-children/.

[13] Fietsersbond. 2023 Is een fietskar veilig? https://www.fietsersbond.nl/nieuws/is-een-fietskar-veilig/.

[14] Veiligheid NL . 2026 Fietskar: veilig vervoeren van 1 tot 2 kinderen. Amsterdam, The Netherlands: Kenniscentrum Letselpreventie VeiligheidNL.

[15] Powell EC , Tanz RR. 2000 Tykes and bikes: injuries associated with bicycle-towed child trailers and bicycle-mounted child seats. Arch. Pediatr. Adolesc. Med. 154, 351–353. (doi:10.1001/archpedi.154.4.351) 10768671

[16] Schwanitz S , Stuff A, Odenwald S. 2020 Exposure of children in a bicycle trailer to whole-body vibration. In Proc. of the 13th Conf. of the International Sports Engineering Association, vol. 49. Chemnitz, Germany: Chemnitz University of Technology. (doi:10.3390/proceedings2020049114)

[17] Vester MEM , Bilo RAC, Loeve AJ, van Rijn RR, van Zandwijk JP. 2019 Modeling of inflicted head injury by shaking trauma in children: what can we learn? Part I: a systematic review of animal models. Forensic Sci. Med. Pathol. 15, 408–422. (doi:10.1007/s12024-019-0082-3) PMC6687679 30828765

[18] van Zandwijk JP , Vester MEM, Bilo RA, van Rijn RR, Loeve AJ. 2019 Modeling of inflicted head injury by shaking trauma in children: what can we learn? Part II: a systematic review of mathematical and physical models. Forensic Sci. Med. Pathol. 15, 423–436. (doi:10.1007/s12024-019-00093-7) PMC6687692 30784025

[19] Hutchinson K , van Zandwijk JP, Vester MEM, Seth A, Bilo RAC, van Rijn RR, Loeve AJ. 2024 Modeling of inflicted head injury by shaking trauma in children: what can we learn? Update to parts I&II: a systematic review of animal, mathematical and physical models. Forensic Sci. Med. Pathol. 21, 366–381. (doi:10.1007/s12024-023-00765-5) 38236351

[20] Schiks LAH , Dankelman J, Loeve AJ. 2020 Thresholds for the assessment of inflicted head injury by shaking trauma in infants: a systematic review. Forensic Sci. Int. 306, 110060. (doi:10.1016/j.forsciint.2019.110060) 31785511

[21] Jones MD , Martin PS, Williams JM, Kemp AM, Theobald P. 2014 Development of a computational biomechanical infant model for the investigation of infant head injury by shaking. Med. Sci. Law 55, 291–299. (doi:10.1177/0025802414564495) 25550310

[22] Hutchinson K , Stray-Pedersen A, Dankelman J, Seth A, Loeve AJ. 2025 Comparing the kinematics related to inflicted head injury between violent shaking of a 6-week-old and a 1-year-old infant surrogate. R. Soc. Open Sci. 12, 251251. (doi:10.1098/rsos.251251) PMC12646764 41307067

[23] Glowinski S , Głowińska A. 2025 Unveiling the abusive head trauma and Shaken Baby Syndrome: a comprehensive wavelet analysis. Biomed. Signal Process. Control 107, 107862. (doi:10.1016/j.bspc.2025.107862)

[24] Song HH , Thoreson WB, Dong P, Shokrollahi Y, Gu L, Suh DW. 2022 Exploring the vitreoretinal interface: a key instigator of unique retinal hemorrhage patterns in pediatric head trauma. Korean J. Ophthalmol. 36, 253–263. (doi:10.3341/kjo.2021.0133) PMC9194735 35527527

[25] Herman BE , Makoroff KL, Corneli HM. 2011 Abusive head trauma. Pediatr. Emerg. Care 27, 65–69. (doi:10.1097/PEC.0b013e31820349db) 21206262

[26] Schiks LAH , Dankelman J, Loeve AJ. 2023 Inflicted head-injury by shaking-trauma in infants: the importance of spatiotemporal variations of the head's rotation center. Sci. Rep. 13, 15226. (doi:10.1038/s41598-023-42373-x) PMC10502057 37709812

[27] Batterbee DC , Sims ND, Becker W, Worden K, Rowson J. 2011 Computational model of an infant brain subjected to periodic motion simplified modelling and Bayesian sensitivity analysis. Proc. Inst. Mech. Eng. H. 225, 1036–1049. (doi:10.1177/0954411911420002) 22292202

[28] Lee M , Melvin J, Ueno K. 1987 Finite element analysis of traumatic subdural hematoma. In Proc. 31st Stapp Car Crash Conf., pp. 29–39. New Orleans, LA: SAE International.

[29] Morison CN . 2002 The dynamics of shaken baby syndrome. PhD thesis, University of Birmingham, UK.

[30] Nadarasa J , Deck C, Meyer F, Raul JS, Willinger R. 2015 Infant eye finite element model to investigate retinal hemorrhages after fall and shaking events. Comput. Methods Biomech. Biomed. Engin. 18, 2016–2017. (doi:10.1080/10255842.2015.1069602) 26230636

[31] Ommaya AK . 1968 Whiplash injury and brain damage. J. Am. Med. Assoc. 204, 285–289. (doi:10.1001/jama.1968.03140170001001) 4967499

[32] Ommaya AK , Gennarelli TA. 1974 Cerebral concussion and traumatic unconsciousness. Brain 97, 633–654. (doi:10.1093/brain/97.1.633) 4215541

[33] Suh DW , Song HH, Mozafari H, Thoreson WB. 2021 Determining the tractional forces on vitreoretinal interface using a computer simulation model in abusive head trauma. Am. J. Ophthalmol. 223, 396–404. (doi:10.1016/j.ajo.2020.06.020) 32663454

[34] Lintern TO , Nash MP, Kelly P, Bloomfield FH, Taberner AJ, Nielsen PMF. 2017 Probabilistic description of infant head kinematics in abusive head trauma. Comput. Methods Biomech. Biomed. Eng. 20, 1633–1642. (doi:10.1080/10255842.2017.1403593) 29160091

[35] Smith DH , Meaney DF. 2002 Roller coasters, g forces, and brain trauma: on the wrong track? J. Neurotrauma 19, 1117–1120. (doi:10.1089/08977150260337921) 12427321

[36] Allen ME , Weir-Jones I, Motiuk DR, Flewin KR, Goring RD, Kobetitch R, Broadhurst A. 1994 Acceleration perturbations of daily living. A comparison to ‘whiplash’. Spine 19, 1285–1290. (doi:10.1097/00007632-199405310-00017) 8073323

[37] Hutchinson K , Bieseman P, Dankelman J, Seth A, Loeve AJ. 2026 Source data of journal article: Violent shaking versus a bicycle trailer ride: Experimental comparison of kinematics in infant injury scenarios. 4TU. ResearchData. Ddataset. (doi:10.4121/bf0d53ae-ace8-4e3c-8f61-30e6f4990ba6.v1)

[38] Hutchinson K , Bieseman P, Dankelman J, Seth A, Loeve AJ. 2026 Supplementary material from: Violent shaking versus a bicycle trailer ride: experimental comparison of kinematics in infant injury scenarios. Figshare. (doi:10.6084/m9.figshare.c.8315018)

