# Supplementary material for: Violent shaking versus a bicycle trailer ride: experimental comparison of kinematics in infant injury scenarios
Source: R Soc Open Sci. 2026 Oct 28;13(10):260279. doi: 10.1098/rsos.260279 (PMC13714421; doi:10.1098/rsos.260279)
Supplement: Supplementary file 1 [file rsos.260279.zip › rsos.260279/rsos260279_si_001.pdf]

Supplementary materials

Supplementary materials accompanying the journal article titled:

“ Violent shaking versus a bicycle trailer ride: Experimental comparison of kinematics in infant injury scenarios”

Authors

Kim Hutchinson [1]
https://orcid.org/0009-0000-5114-8185

Puck Bieseman [1]
https://orcid.org/0009-0005-1255-5550

Jenny Dankelman [1]
https://orcid.org/0000-0003-3951-2129

Ajay Seth [1]
https://orcid.org/0000-0003-4217-1580

Arjo J. Loeve [1, 2]
https://orcid.org/0000-0002-4449-9582

[1] Department of BioMechanical Engineering, Faculty of Mechanical Engineering, Delft University of Technology, Delft, The Netherlands.

[2] Co van Ledden Hulsebosch Centre for Forensic Science and Medicine, Amsterdam, The Netherlands.

* Corresponding author: (a.j.loeve@tudelft.nl)

APPENDIX A – ADDITIONAL EXPLORATIVE 3-BUMP TRIALS

As mentioned in the primary journal article, an additional set of explorative experiments were conducted for a subset of the main test conditions, except with an extra bump in the track, to explore if a resonance frequency would be reached that would exacerbate head accelerations. To summarise, this limited additional test did not result in a resonance frequency of the head movement being reached. That is, none of the trials resulted in the head's linear and/or angular accelerations consistently increasing in magnitude with consecutive bumps. In several trials, the trailer flew over the additional bump entirely. The results from this test are included in the data set accompanying the article, but as no additional insights were gained from these tests, they were not included in the article.

APPENDIX B – FIELD SURVEY OF BUMPS IN DELFT

Table B1 lists several road irregularities with measured height (top to road), slope length (face length, or the run), and slope angle (face angle, degrees relative to the horizontal). The field observations spanned slopes between 13° and 63°, and heights between 2 and 15 centimetres. A standard bump was derived (with standard slope and size): 26°, 50mm tall (bump type C). Other bump profiles with different slope angles were derived by varying the slope length (to realize ~14° and ~45° at 50 mm height). Bump profiles with different sizes were derived by varying the height, without varying the slope (heights 25 and 100mm). The resulting bump types were A (25 mm, 26°), B (50 mm, 45°), C (50 mm, 26°), D (50 mm, 14°), E (100 mm, 26°).

Table B1 - Field survey measurements of bumps in Delft, The Netherlands

Description	Height (cm)	Length (cm)	Slope (deg)
**Sewer lid**	8	35	13
**Curb with dirt ramp**	10	15	34
**Sidewalk curb**	10	75	8
**Steep sidewalk curb**	10	14	35
**Steep sidewalk curb including lowered ground***	11	14	38
**Sewer lid city centre****	2	5	22
**Concrete slabs**	2	1	63
**Sidewalk curb**	15	50	17
**Sewer lid**	8	35	13
**Curb with dirt ramp**	10	15	34
**Sidewalk curb**	10	75	8

Notes:

**Many curb ramps started from a slightly lowered approach surface, which effectively increased the face angle and step height perceived by the cyclist.*

***Sewer lids were often set below the grade of the road. This depended on the surrounding paving. Effectively, the height and/or run could be smaller or larger than the lid itself.*

APPENDIX C – SCHEMATIC TOPVIEW OF EXPERIMENTAL SETTING

A schematic top view of the experimental setup is provided in Figure D1. At a framerate of 500 fps the PrimeX13 cameras have a field of view of 42° horizontally and 34° vertically.

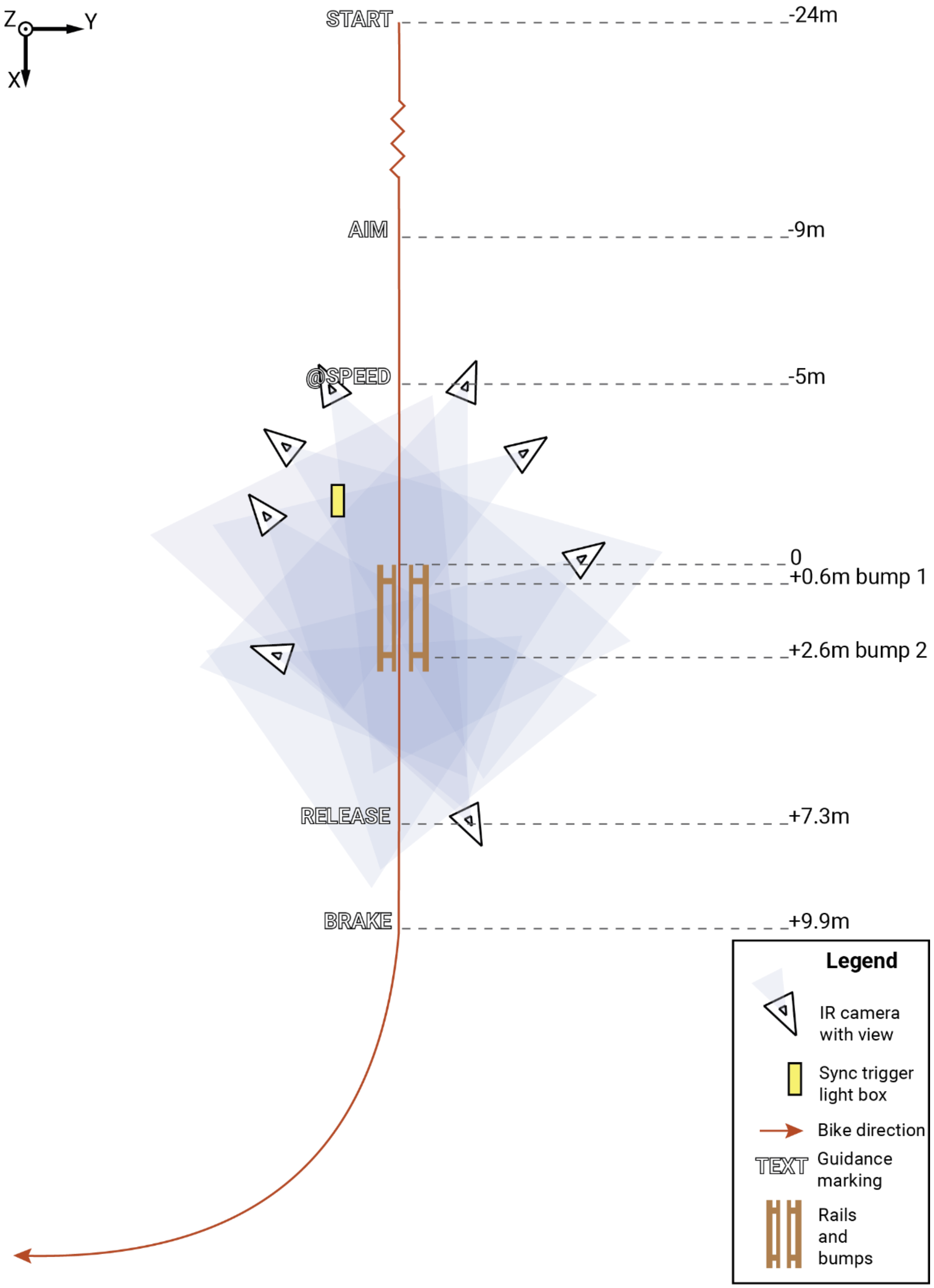

Figure D1 – Schematic top view of the experimental parkour. Infrared motion capture cameras (white triangle, black outline) with their fields of view (blue triangles) indicate the covered volume of the cameras. The location of the synchronisation trig

APPENDIX D – TIRE PRESSURE MAINTENANCE PROTOCOL

A tire pump (product number 41120034, HEMA, Amsterdam, the Netherlands) was used to keep the tires of the bicycle at a pressure of 4 bar (in accordance with the recommended pressure stated on its outer tires), and those on the trailer at 2.3 bar (in accordance with the recommended pressure between 2.1 and 2.4 bar stated on the warning label on the trailer's inner cover). The tire pressures were checked before the first experiment of each day and after every ninth trial throughout the day.

APPENDIX E – CUSTOM STEEL SEAT

The custom trailer seat was made of a 2mm thick steel sheet that was laser cut and then bent to a 60 degree angle. The schematic drawing of the dimensions of the sheet and its cutting line are depicted in Figure E1.

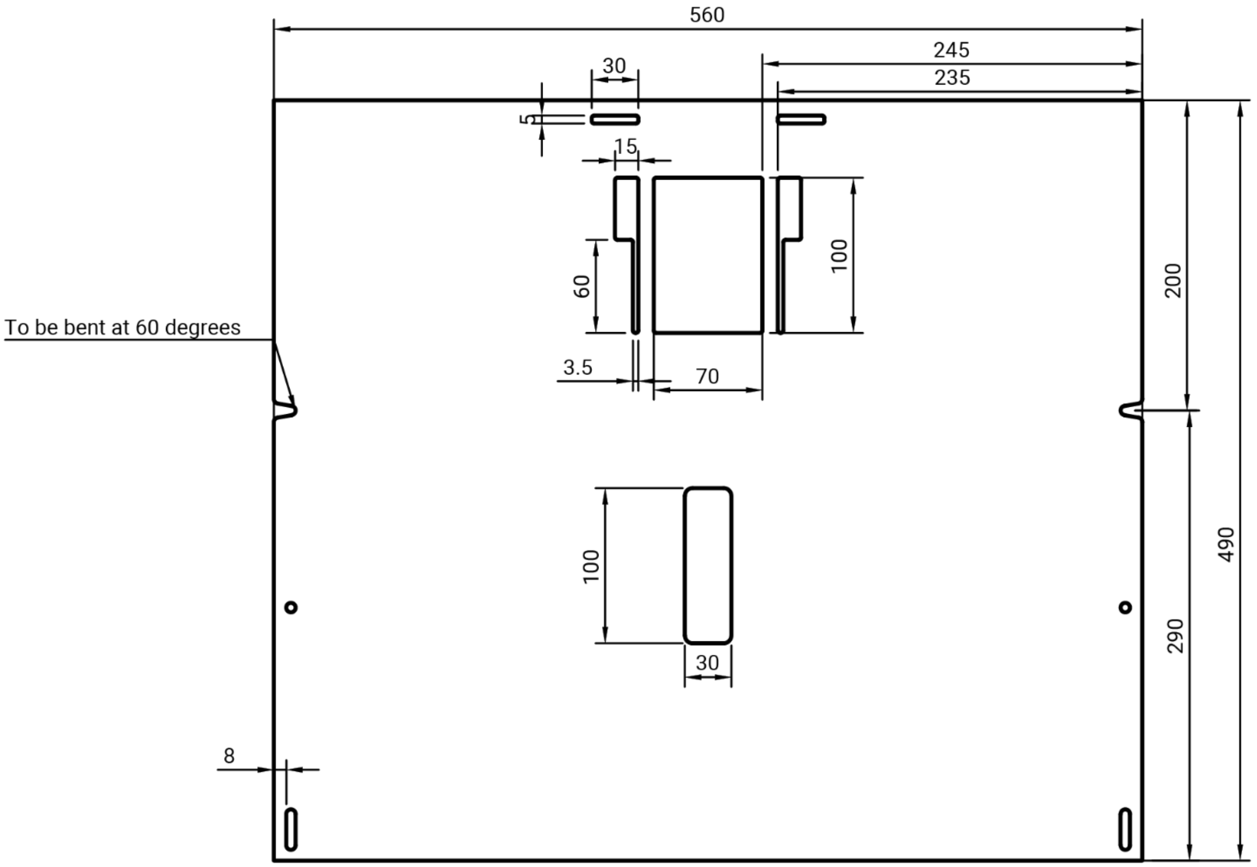

Figure E1 – Schematic drawing of the dimensions (mm) of the cutting lines of the custom trailer sheet.

APPENDIX F – DUMMY AND BICYCLE INSTRUMENTATION

Overview

The Q0 instrumentation was powered with a 12V battery and a custom-made power supply. The sync trigger system was directly powered by the 12V battery. Signals from the instrumentation and the trigger system were directed to the DAQ, which was connected to a laptop. Figure F1 shows the overview of all the components.

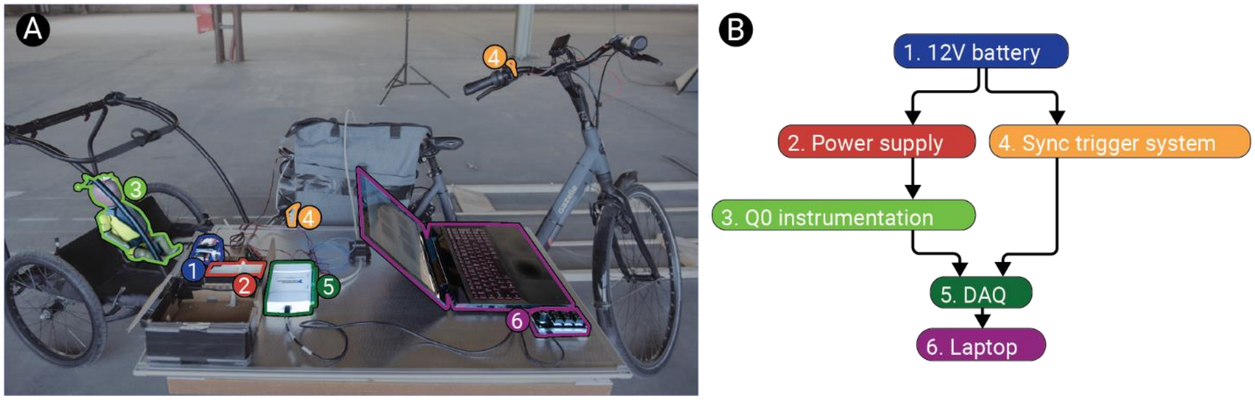

Figure F1 – Overview of sensor instrumentation connections. A: An overview of the objects that are linked to the sensor system. Each number contains an object. B: Shows the names of those objects and the order in which they are plugged.

Power supply

The power supply uses a 12V 1.3Ah UltraCell UL1.3-12 VRLA AGM Lead Battery with T1 terminals and has output voltages of 5V for the gyroscope and 3.3V for the accelerometers, respectively (Figure F2 , F3).

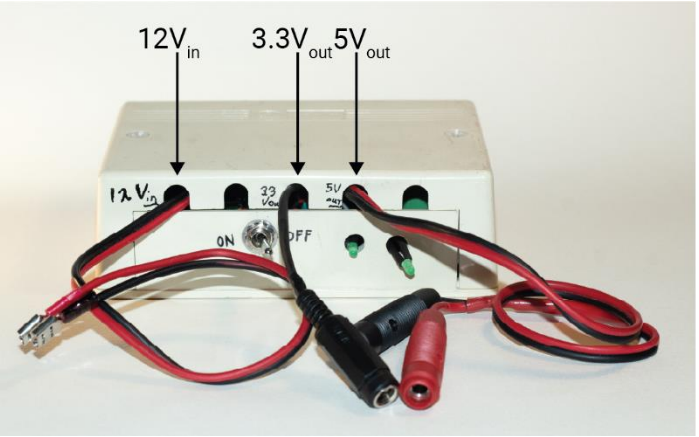

Figure F2 – The power supply with wires for 12V input and 3.3V and 5V output.

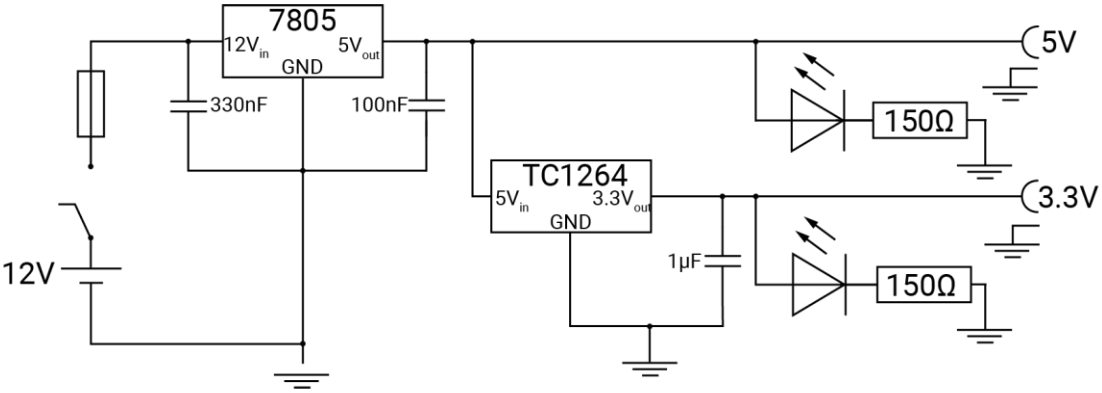

Figure F3 – Circuit of the power supply according to IEC 60617 symbol standards.

Data synchronisation trigger system

The synchronisation trigger system consists of a circuit in a box (Figure F4A), a manual trigger button (Figure F4B) and a light source (Figure F4C-D). The light source was an LED construction lamp with a reflective box around it to only shine light in the desired direction, so that the system would not be triggered too early or too late. The circuit is shown in Figure F5.

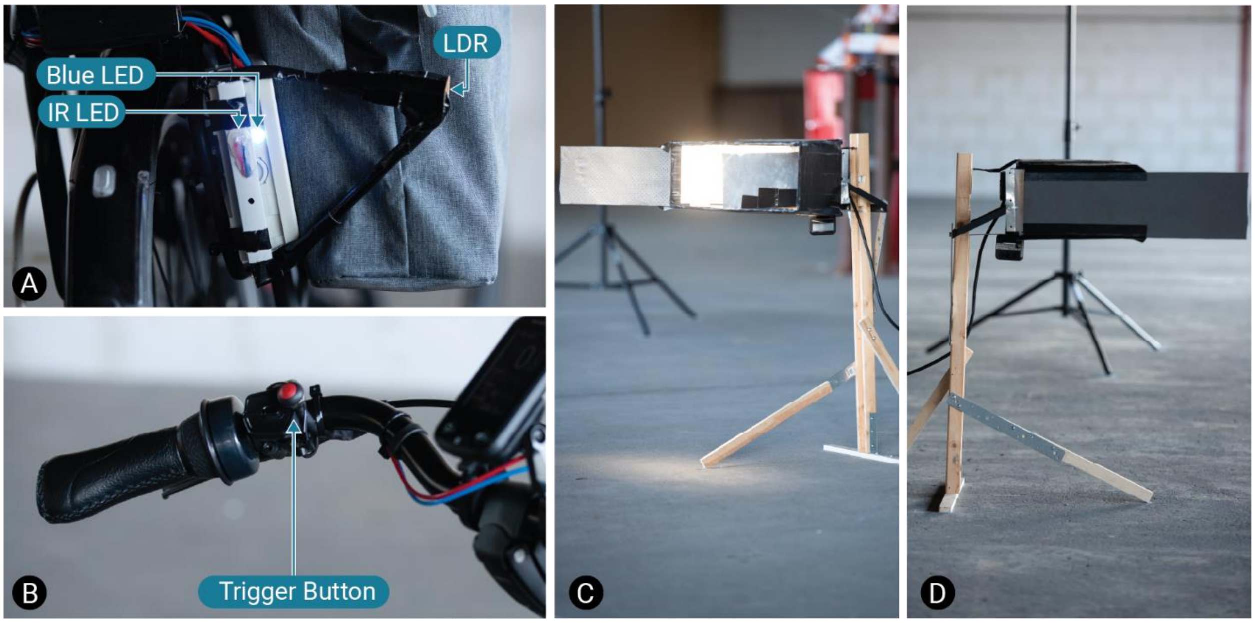

Figure F4 – Overview of the different components belonging to the synchronisation trigger system. A: Box containing the LDR sensor and circuit to provide output trigger signals in the form of an 850 nm IR LED and a 5V voltage spike. The blue LED functions as an indicator light to show that the circuit was working, since IR light is not visible to the human eye. B: The manual trigger button that could trigger the synchronisation signals in the case the light trigger did not work. C: Front view of the light box that would trigger the LDR. D: Back view of the light box.

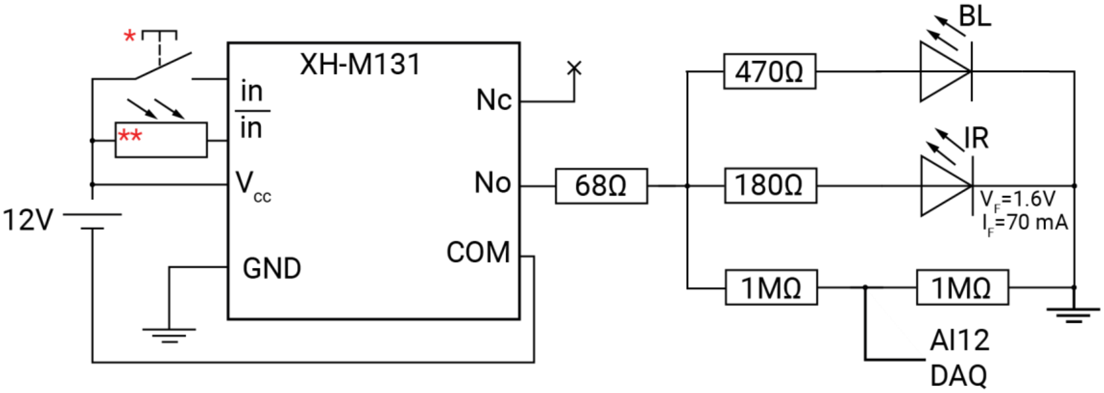

*Figure F5 – Circuit of the synchronisation trigger system according to IEC 60617 symbol standards. * NO push button with automatic return, as in Figure D4B. ** LDR is part of the XH-M131, as indicated in Figure D4A.*

Computation of Q0 head and torso local reference frame

The orientation of the local reference frames of the head and torso and the positions of the respective origins in the global reference frame were computed for each frame in the motion capture data. This was done based on the positions of the head and torso reflective marker clusters, also referred to as 'tracking marker cluster' (Figure F6). For this, separate stationary motion capture takes were recorded which were used to determine the orientation of the marker clusters in the local reference frames of the head and torso, and the offset between the clusters and the local reference frame using known 'reference markers' (Figure F6) to indicate the locations and orientations of the local reference frames. The recordings and computations were performed as described in the Supplementary Material of Hutchinson et al (2025), with a slight adjustment step four: The recording of the reference measurements. In this step, it was described that in the recorded take both the four tracking markers as well as the reference markers should be clearly visible. In our case, the reference markers were occluded. Therefore, only the complete set of tracking markers and one reference marker was mounted during each recording, thereby obtaining a separate recording for each tracking marker.

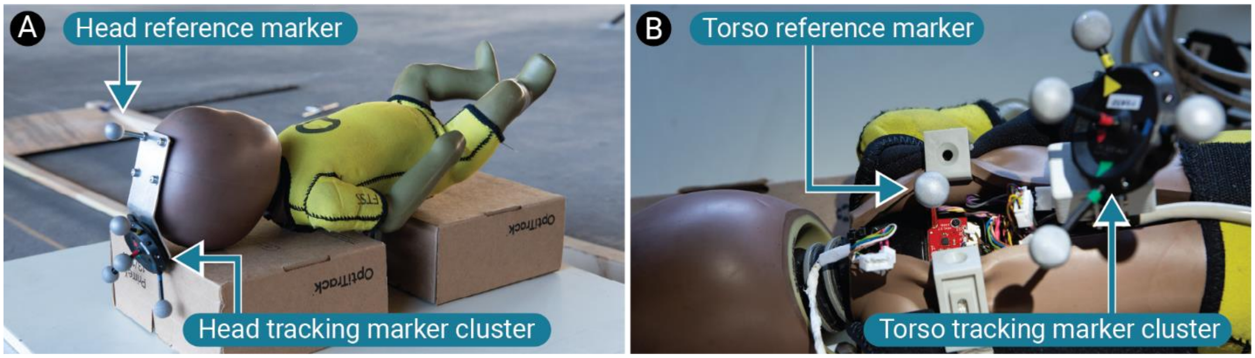

Figure F6 – Picture of the setup for recording a motion capture reference recording by recording each reference marker separately. A: Setup for recording one of the head reference markers. B: Setup for recording one of the torso reference markers.

APPENDIX G – MOTION CAPTURE GAP FILLING PROTOCOL

The workflows for gap-filling of missing frames for the head and torso were similar, but not identical, because the head was expected to move around a more and more erratically than the torso, which closely matched the trailer trajectory due to the torso being tightly strapped to the seat. Gap filling was mainly necessary when the head, torso, and trailer objects entered or left the scene for all trials, and in less than half of the trials small gaps (less than a dozen consecutive frames) occurred during trailer bouncing. The following approaches were used for gap filling:

- **Torso marker gap filling**: Gaps of torso marker trajectories were filled if data in no more than 100 consecutive frames were missing. If one or two markers of the marker cluster of four were missing, pattern-based interpolation was used based on the trajectories of the remaining three or two markers. If two markers were missing and the trailer was in-flight, and pattern-based interpolation did not yield expected results, cubic interpolation was applied.

- **Head marker gap filling**: Gaps of the head marker trajectories were filled if data in no more than 25 consequent frames were missing. If only one marker of the cluster of four was missing, pattern-based interpolation was done based on the remaining three markers. If two markers were missing and the trailer was in-flight, pattern-based interpolation was done based on the remaining two markers.

There were two trials (trials 52 and 53) for which exceptions were made. In these trials, three torso markers were missing. To solve this, one of the marker gaps was cubically interpolated, and then the other two gaps were filled using pattern-based interpolation based on the two marker trajectories that existed now.

APPENDIX H – Determining bounce window

The end of the *first bounce* window was determined as follows:

1. Find the timestamp of the trailer landing after the first flight by computing the first local minimum of the Fc z-trajectory after the first bump (Figure H1 L1).
2. Starting from trailer landing, find the first timestamp of when the neck flexion goes from negative to positive.
3. Add an arbitrary margin of 50 frames (at sensor sample rate) to ensure that no peaks are missed.

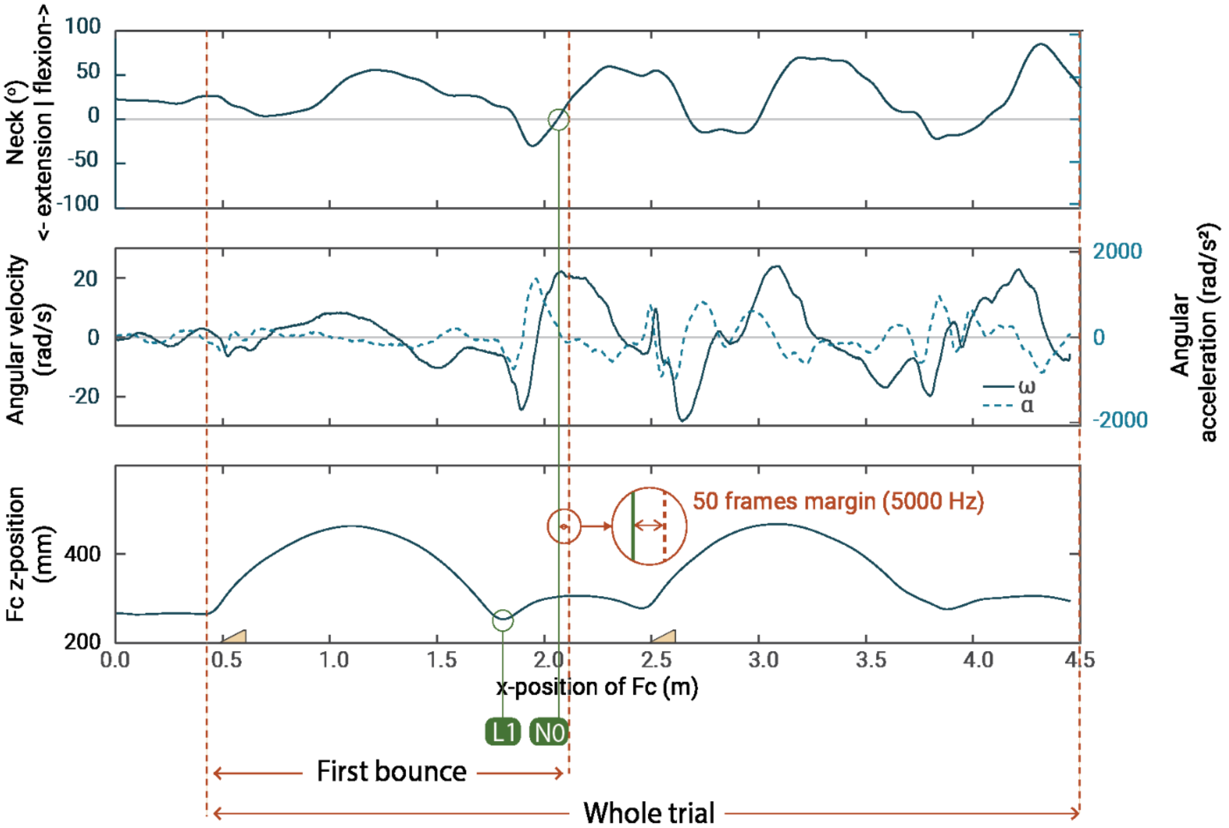

Figure H1 – Depiction of the range of the first bounce and the whole trial. From top to bottom, the angular velocity and acceleration, neck extension and flexion, and the z-position of trailer marker Fc are plotted against the x-position of marker Fc. L1 indicates the time when the trailer lands for the first time, with a green circle drawn around the first negative peak of the Fc z-trajectory. N0 indicates the first time when the sagittal neck angle sign of the Q0 surrogate becomes positive again after L1. The end of the first bounce range is set at a somewhat arbitrary value of 50 sensor data frames (5000 Hz) after L1 to provide margin and not miss any potential angular acceleration peaks. The start of both ranges was located 50 mm before the first bump started to provide margin at the beginning of the ranges. The data in this figure was retrieved from trial 47, and the method for obtaining the ranges for the first bounce and the whole trial was done using the same method as described here.

APPENDIX I – RESULTS & STATISTICS

Outcomes whole trial

Table I1 – Outcomes over the whole trial: Median (MD) values, standard deviations (SD), and interquartile range (IQR) of all outcomes, per testing condition. Abbreviations are lin. (linear), vel. (velocity), acc. (acceleration). Linear acceleration concerns the resultant sagittal linear acceleration.

Testing condition	Bump type	Velocity	Slope	Height	N		Neck flexion (°)	Neck extension (°)	Lin. acc. head (m/s²)	Ang. vel. head (rad/s)	Ang. acc. head (rad/s²)	Lin. acc. torso (m/s²)	Ang. vel. Torso (rad/s)	Ang. acc. head (rad/s²)	IROR (m)
All					98	MD	62.96	-26.61	151.78	24.88	1302.58	102.83	6.96	469.22	0.65
						Q1	57.39	-31.22	133.10	21.75	1098.58	86.69	6.00	401.44	0.54
						Q3	67.43	-19.49	173.33	25.73	1477.93	126.30	7.33	535.15	0.94
						IQR	10.05	11.72	40.23	4.82	379.35	39.61	2.00	133.71	0.39
1	A	10	26	25	9	MD	61.79	-18.86	141.03	24.95	1239.17	92.75	7.37	471.19	0.58
						Q1	59.85	-22.37	133.77	24.10	1166.30	85.29	6.93	451.75	0.54
						Q3	65.89	-18.00	145.53	25.01	1308.49	98.93	7.44	477.75	0.62
						IQR	6.04	4.36	11.76	1.46	142.19	13.63	0.59	25.99	0.08
2	A	15	26	25	9	MD	66.87	-20.48	150.98	26.65	1312.74	104.16	7.63	530.96	0.62
						Q1	62.16	-21.84	133.30	24.47	1209.78	98.40	6.89	506.49	0.60
						Q3	69.80	-18.98	179.34	27.04	1392.18	142.84	7.78	673.29	0.68
						IQR	7.65	2.86	46.03	2.36	182.40	44.44	2.10	166.79	0.08
3	A	20	26	25	9	MD	63.15	-12.77	121.47	22.53	999.79	80.62	6.18	338.84	1.09
						Q1	60.36	-14.43	105.57	21.22	837.39	70.92	4.87	333.02	1.03
						Q3	65.60	-9.10	132.33	22.66	1075.48	83.56	6.36	397.30	1.15
						IQR	5.24	5.33	26.76	2.33	238.09	12.63	1.58	64.28	0.12
4	B	10	45	50	9	MD	65.19	-31.32	108.60	24.26	1223.28	92.96	8.39	438.93	0.35
						Q1	64.04	-32.22	105.07	22.40	1168.77	84.93	6.60	368.40	0.31
						Q3	67.46	-27.95	161.61	24.54	1463.15	111.99	8.53	469.00	0.38
						IQR	3.42	4.27	56.54	2.52	294.38	27.06	2.19	100.60	0.07
5	C	10	26	50	8	MD	65.69	-30.73	146.57	26.88	1419.03	105.29	6.58	423.90	0.44
						Q1	61.93	-31.11	123.85	22.22	1335.08	91.14	5.86	355.07	0.42
						Q3	71.79	-29.15	162.43	27.35	1496.01	114.96	7.13	440.07	0.56
						IQR	9.86	1.96	38.58	4.49	160.93	23.82	2.30	84.99	0.15
6	C	15	26	50	9	MD	61.83	-32.76	253.46	29.27	1496.35	138.62	7.00	663.46	0.70
						Q1	59.82	-34.29	188.05	25.34	1339.78	132.92	6.79	525.99	0.67
						Q3	63.75	-31.47	284.42	29.77	1880.39	162.60	7.51	710.54	0.74
						IQR	3.93	2.82	96.37	5.06	540.61	29.68	1.48	184.55	0.07
7	C	20	26	50	9	MD	56.90	-29.45	257.39	29.79	1933.54	160.89	11.36	760.26	1.20
						Q1	51.63	-32.59	233.78	28.63	1828.08	156.63	9.66	698.62	1.04
						Q3	64.04	-28.16	262.89	29.90	1993.17	164.67	11.48	808.26	1.29
						IQR	12.41	4.43	29.11	2.19	165.09	8.04	2.21	109.63	0.25
8	D	10	14	50	9	MD	64.98	-17.76	149.67	19.40	1031.80	86.22	6.52	459.37	0.65
						Q1	57.22	-20.75	134.07	18.88	1007.83	82.64	5.64	432.03	0.59
						Q3	68.25	-16.60	150.62	19.70	1068.99	88.61	6.63	474.68	0.70
						IQR	11.03	4.15	16.55	1.57	61.16	5.97	1.02	42.65	0.10
9	D	15	14	50	9	MD	57.28	-27.88	148.39	23.81	1316.78	101.23	6.48	478.65	0.67
						Q1	55.85	-30.78	133.27	23.19	1274.12	98.76	5.20	370.27	0.64
						Q3	59.54	-25.02	154.70	24.15	1361.33	107.31	7.22	529.73	0.71
						IQR	3.68	5.76	21.43	1.54	87.22	8.55	2.61	159.45	0.07
10	D	20	14	50	9	MD	55.48	-29.90	163.37	25.91	1465.34	121.08	6.94	504.00	1.23
						Q1	53.91	-32.72	148.74	24.49	1377.86	117.10	6.00	404.06	1.05
						Q3	56.64	-28.72	183.06	25.95	1648.83	123.65	6.96	537.29	1.24
						IQR	2.73	4.00	34.32	1.30	270.96	6.55	1.05	133.24	0.19
11	E	10	26	100	9	MD	68.60	-20.70	161.87	22.39	1037.70	96.08	6.30	455.78	0.49
						Q1	64.76	-24.24	155.65	18.59	837.76	87.74	5.42	407.98	0.46
						Q3	74.28	-17.00	168.04	23.28	1265.08	110.72	6.41	488.01	0.52
						IQR	9.52	7.24	12.40	5.94	427.32	22.98	2.47	80.03	0.06

Outcomes first bounce

Table I2 – Outcomes over the first bounce: Median (MD) values, standard deviations (SD), and interquartile range (IQR) of all outcomes, per testing condition. Abbreviations are lin. (linear) and acc. (acceleration). Linear acceleration concerns the resultant sagittal linear acceleration.

Testing condition	Bump type	Velocity	Slope	Height	N		Neck flexion (°)	Neck extension (°)	Lin. acc. head (m/s²)	Ang. vel. head (rad/s)	Ang. acc. head (rad/s²)	Lin. acc. torso (m/s²)	Ang. vel. Torso (rad/s)	Ang. acc. head (rad/s²)	IROR (m)
All					98	MD	51.57	-23.16	138.56	24.88	1185.83	93.90	6.96	384.60	0.74
						Q1	47.24	-30.15	114.89	21.75	957.61	79.95	6.00	328.02	0.62
						Q3	56.70	-16.93	164.88	25.73	1413.59	118.34	7.33	481.72	1.05
						IQR	9.46	13.22	49.99	4.82	455.98	38.39	2.00	153.70	0.43
1	A	10	26	25	9	MD	44.95	-18.19	136.79	24.95	1185.19	79.10	7.37	391.80	0.76
						Q1	44.52	-19.26	91.82	24.10	1115.60	67.99	6.93	358.09	0.70
						Q3	47.57	-15.89	143.29	25.01	1234.96	84.13	7.44	441.87	0.79
						IQR	3.06	3.37	51.47	1.46	119.36	16.14	0.59	83.78	0.09
2	A	15	26	25	9	MD	53.25	-20.27	135.10	26.65	1186.46	101.46	7.63	471.64	0.62
						Q1	51.08	-20.72	121.51	24.47	1011.05	76.15	6.89	398.85	0.60
						Q3	55.85	-18.81	145.66	27.04	1292.06	103.28	7.78	484.51	0.66
						IQR	4.77	1.91	24.15	2.36	281.00	27.13	2.10	85.67	0.06
3	A	20	26	25	9	MD	54.11	-12.77	114.89	22.53	851.70	68.04	6.18	336.80	1.31
						Q1	48.16	-14.43	99.12	21.22	714.38	52.87	4.87	263.14	1.19
						Q3	57.41	-8.55	129.56	22.66	1075.48	81.14	6.36	352.63	1.37
						IQR	9.26	5.88	30.44	2.33	361.10	28.27	1.58	89.48	0.18
4	B	10	45	50	9	MD	54.50	-25.42	93.85	24.26	719.26	74.93	8.39	256.66	0.50
						Q1	53.64	-27.62	85.77	22.40	621.89	73.75	6.60	236.36	0.46
						Q3	55.91	-22.88	96.90	24.54	954.49	78.18	8.53	266.51	0.54
						IQR	2.27	4.75	11.13	2.52	332.61	4.43	2.19	30.14	0.08
5	C	10	26	50	8	MD	47.25	-30.73	132.87	26.88	1419.03	102.75	6.58	339.06	0.66
						Q1	46.94	-31.11	119.54	22.22	1200.13	84.51	5.86	289.69	0.58
						Q3	50.21	-29.15	141.80	27.35	1496.01	114.96	7.13	375.77	0.73
						IQR	3.27	1.96	22.26	4.49	295.88	30.46	2.30	86.08	0.15
6	C	15	26	50	9	MD	58.76	-32.76	253.46	29.27	1496.35	138.62	7.00	651.29	0.71
						Q1	56.73	-34.29	180.29	25.34	1287.97	132.11	6.79	408.41	0.64
						Q3	59.64	-31.47	284.42	29.77	1880.39	162.60	7.51	694.78	0.72
						IQR	2.91	2.82	104.12	5.06	592.42	30.49	1.48	286.37	0.08
7	C	20	26	50	9	MD	47.39	-29.45	257.39	29.79	1933.54	160.89	11.36	760.26	1.27
						Q1	46.48	-32.59	233.78	28.63	1828.08	156.63	9.66	698.62	1.16
						Q3	51.91	-28.16	262.89	29.90	1993.17	164.67	11.48	808.26	1.49
						IQR	5.43	4.43	29.11	2.19	165.09	8.04	2.21	109.63	0.33
8	D	10	14	50	9	MD	43.37	-17.76	132.79	19.40	1009.11	82.82	6.52	380.68	0.81
						Q1	42.42	-19.57	117.96	18.88	1002.72	79.53	5.64	336.41	0.71
						Q3	45.13	-16.60	145.71	19.70	1033.07	86.00	6.63	466.27	0.89
						IQR	2.71	2.97	27.76	1.57	30.35	6.48	1.02	129.87	0.18
9	D	15	14	50	9	MD	49.62	-26.61	128.94	23.81	1289.00	100.83	6.48	348.89	0.83
						Q1	47.42	-29.82	114.93	23.19	1260.71	96.52	5.20	325.34	0.77
						Q3	52.14	-24.26	141.08	24.15	1341.56	105.78	7.22	380.18	0.90
						IQR	4.72	5.55	26.15	1.54	80.85	9.26	2.61	54.84	0.14
10	D	20	14	50	9	MD	53.07	-29.90	163.37	25.91	1465.34	121.08	6.94	504.00	1.35
						Q1	51.32	-32.72	148.74	24.49	1377.86	117.10	6.00	404.06	1.23
						Q3	56.46	-28.72	183.06	25.95	1648.83	123.65	6.96	537.29	1.47
						IQR	5.14	4.00	34.32	1.30	270.96	6.55	1.05	133.24	0.24
11	E	10	26	100	9	MD	60.50	-9.65	161.75	22.39	570.39	86.59	6.30	405.10	0.59
						Q1	59.22	-11.80	155.65	18.59	515.53	81.97	5.42	371.71	0.55
						Q3	62.14	-5.18	165.28	23.28	649.12	92.87	6.41	430.44	0.62
						IQR	2.92	6.62	9.63	5.94	133.59	10.90	2.47	58.73	0.06

Statistical test results – normal distribution

Tests were conducted to determine whether the distributions of outcome measures differed significantly from normal for the whole trial (Table I3) and the first bounce (Table I4).

*Table I3 – P-values for Anderson-Darling tests for outcome measures over the whole trial, per testing condition. Null-hypothesis: the test distribution is drawn from a standard normal distribution. *Reject null hypothesis, significance level p<0.05 (two-tailed).*

Testing condition	Bump type	Velocity	Slope	Height	N		Neck flexion	Neck extension	Lin. acc. head	Ang. acc. head	Lin. acc. torso	Ang. acc. torso	IROR
All					98	0.182	<0.0005*	<0.0005*	0.013*	0.001*	<0.0005*	<0.0005*	All
1	A	10	26	25	9	0.756	0.24	0.001*	<0.0005*	0.421	<0.0005*	0.111	1
2	A	15	26	25	9	0.274	0.275	0.187	<0.0005*	0.215	0.109	0.055	2
3	A	20	26	25	9	0.591	0.427	0.561	0.006*	0.057	0.045*	0.807	3
4	B	10	45	50	9	0.656	0.148	0.123	0.764	0.143	0.538	0.698	4
5	C	10	26	50	8	0.12	0.064	0.721	0.617	0.85	0.113	0.25	5
6	C	15	26	50	9	0.411	0.922	0.148	0.324	0.247	0.233	0.727	6
7	C	20	26	50	9	0.356	0.719	0.493	0.819	0.135	0.936	0.788	7
8	D	10	14	50	9	0.443	0.079	0.052	0.030*	0.004*	0.31	0.709	8
9	D	15	14	50	9	0.001*	0.935	0.809	<0.0005*	0.372	0.576	0.841	9
10	D	20	14	50	9	<0.0005*	0.351	0.85	0.55	0.878	0.666	0.083	10
11	E	10	26	100	9	0.471	0.218	0.002*	0.925	0.032*	0.603	0.121	11

*Table I4 – P-values for Anderson-Darling tests for outcome measures over the first bounce, per testing condition. Null-hypothesis: the test distribution is drawn from a standard normal distribution. *Reject null hypothesis, significance level p<0.05 (two-tailed).*

Testing condition	Bump type	Velocity	Slope	Height	N		Neck flexion	Neck extension	Lin. acc. head	Ang. acc. head	Lin. acc. torso	Ang. acc. torso	IROR
All					98	0.182	<0.0005*	<0.0005*	0.013*	0.001*	<0.0005*	<0.0005*	All
1	A	10	26	25	9	0.756	0.24	0.001*	<0.0005*	0.421	<0.0005*	0.111	1
2	A	15	26	25	9	0.274	0.275	0.187	<0.0005*	0.215	0.109	0.055	2
3	A	20	26	25	9	0.591	0.427	0.561	0.006*	0.057	0.045*	0.807	3
4	B	10	45	50	9	0.656	0.148	0.123	0.764	0.143	0.538	0.698	4
5	C	10	26	50	8	0.12	0.064	0.721	0.617	0.85	0.113	0.25	5
6	C	15	26	50	9	0.411	0.922	0.148	0.324	0.247	0.233	0.727	6
7	C	20	26	50	9	0.356	0.719	0.493	0.819	0.135	0.936	0.788	7
8	D	10	14	50	9	0.443	0.079	0.052	0.030*	0.004*	0.31	0.709	8
9	D	15	14	50	9	0.001*	0.935	0.809	<0.0005*	0.372	0.576	0.841	9
10	D	20	14	50	9	<0.0005*	0.351	0.85	0.55	0.878	0.666	0.083	10
11	E	10	26	100	9	0.471	0.218	0.002*	0.925	0.032*	0.603	0.121	11

Statistical test results – experiment effects

Tests were conducted to determine whether the experimental conditions (bump height and slope, trailer velocity) had a significant effect on the outcome measures. The results are presented in Table I5

*Table I5 – Overall asymptotic p-value and H0 decision of independent-samples Kruskal-Wallis (KW) tests between three bump slopes (testing conditions 8 (14°), 5 (26°), 4 (45°)), three bump heights (testing conditions 1 (25mm), 5 (50mm), 11 (100mm)), and three trailer speeds (testing conditions 5 (10km/h), 6 (15km/h), 7 (15km/h)). The null hypothesis (H0) was that the distribution of the outcome is the same across different values of the independent variable (bump slope, bump height, or trailer speed). Bonferroni-adjusted p-values of the Dunn's post hoc tests are also given for the groups where the H0 hypothesis of the Kruskal-Wallis test was rejected. The significance level is 0.050. *p<0.050.*

		**Test**	**Neck flexion**	**Neck extension**	**Lin. acc. head**	**Ang. vel. head**	**Ang. acc. head**	**Lin. acc. torso**	**Ang. vel. torso**	**Ang. acc. torso**	**IROR**
**Slope**	**Post Hoc**	14°-26°	0.065	0.0*	1	0.005	0.046*	0.247	1	0.83	0.177
		26°-45°	0.134	0.247	0.006*	0.001	0.0*	0.025*	0.03	0.018*	0.088
		14°-45°	0.0*	0.035*	0.005*	1	0.207	1	0.062	0.0*	0.0*
	**KW**	Asymptotic sign.	<.001	<.001	0.001	<.001	<.001	0.029	0.017	<.001	<.001
		Decision H0 KW	*Reject*	*reject*	*reject*	*reject*	*reject*	*reject*	*reject*	*reject*	*reject*
**Height**	**Post Hoc**	25mm-50mm	0.928	0.052	1	0.902	0.493	0.023*	0.061	*N/A*	0.106
		50mm-100mm	0.009*	0.0*	0.003*	0.001	0.0*	0.791	1	*N/A*	0.463
		25mm-100mm	0.0*	0.062	0.003*	0.017	0.018*	0.327	0.068	*N/A*	0.001*
	**KW**	Asymptotic sign.	<0.001	<0.001	<0.001	<0.001	<0.001	0.026	0.028	0.057	0.001
		Decision H0 KW	*reject*	*reject*	*reject*	*reject*	*reject*	*reject*	*reject*	*retain*	*reject*
**Speed**	**Post Hoc**	10-15km/h	0.002*	0.067	0.002*	0.148	1	0.021*	0.245	0.055	1
		15-20km/h	0.001*	0.062	1	1	0.035*	0.619	0.026	0.193	0.001*
		10-20km/h	1	1	0.001*	0.011	0.002*	0*	0*	0*	0.002*
	**KW**	Asymptotic sign.	<0.001	0.028	<0.001	0.013	0.002	<0.001	<0.001	<0.001	<0.001
		Decision H0 KW	*reject*	*reject*	*reject*	*reject*	*reject*	*reject*	*reject*	*reject*	*reject*

Comparison of trailer velocities

The cyclist monitored their velocity during the trail with the speedometer mounted to the bike. To check whether the trailer's velocity matched the intended velocity, the mean velocity in range of the "scene"(x=0 – x=4.5 m) was computed for each trial and plotted in Figure I1. There was no overlap in velocity between the groups.

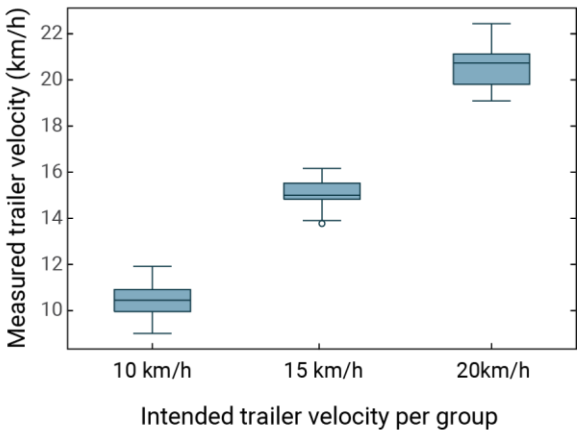

Figure I1 – The mean trailer velocity in range of the "scene"(x=0 – x=4.5 m) was plotted for each intended velocity per experimental condition. No overlap in velocity between groups was found.

		Test	Neck flexion (°)	Neck extension (°)	Lin. acc. head (m/s²)	Ang. acc. head (rad/s²)	Lin. acc. torso (m/s²)	Ang. acc. torso (rad/s²)	IROR (m)
**Slope**	**Post Hoc**	14°-26°	0.065	0.0*	1	0.046*	0.247	0.83	0.177
		26°-45°	0.134	0.247	0.006*	0.0*	0.025*	0.018*	0.088
		14°-45°	0.0*	0.035*	0.005*	0.207	1	0.0*	0.0*
	**KW**	Asymptotic sign.	<.001	<.001	0.001	<.001	0.029	<.001	<.001
		Decision H0 KW	*Reject*	*reject*	*reject*	*reject*	*reject*	*reject*	*reject*
**Height**	**Post Hoc**	25mm-50mm	0.928	0.052	1	0.493	0.023*	*N/A*	0.106
		50mm-100mm	0.009*	0.0*	0.003*	0.0*	0.791	*N/A*	0.463
		25mm-100mm	0.0*	0.062	0.003*	0.018*	0.327	*N/A*	0.001*
	**KW**	Asymptotic sign.	<0.001	<0.001	<0.001	<0.001	0.026	0.057	0.001
		Decision H0 KW	*reject*	*reject*	*reject*	*reject*	*reject*	*retain*	*reject*
**Speed**	**Post Hoc**	10-15km/h	0.002*	0.067	0.002*	1	0.021*	0.055	1
		15-20km/h	0.001*	0.062	1	0.035*	0.619	0.193	0.001*
		10-20km/h	1	1	0.001*	0.002*	0*	0*	0.002*
	**KW**	Asymptotic sign.	<0.001	0.028	<0.001	0.002	<0.001	<0.001	<0.001
		Decision H0 KW	*reject*	*reject*	*reject*	*reject*	*reject*	*reject*	*reject*

Peak duration

The duration and amplitude of head angular velocity and acceleration peaks for two typical trials are indicated in Figure I2.

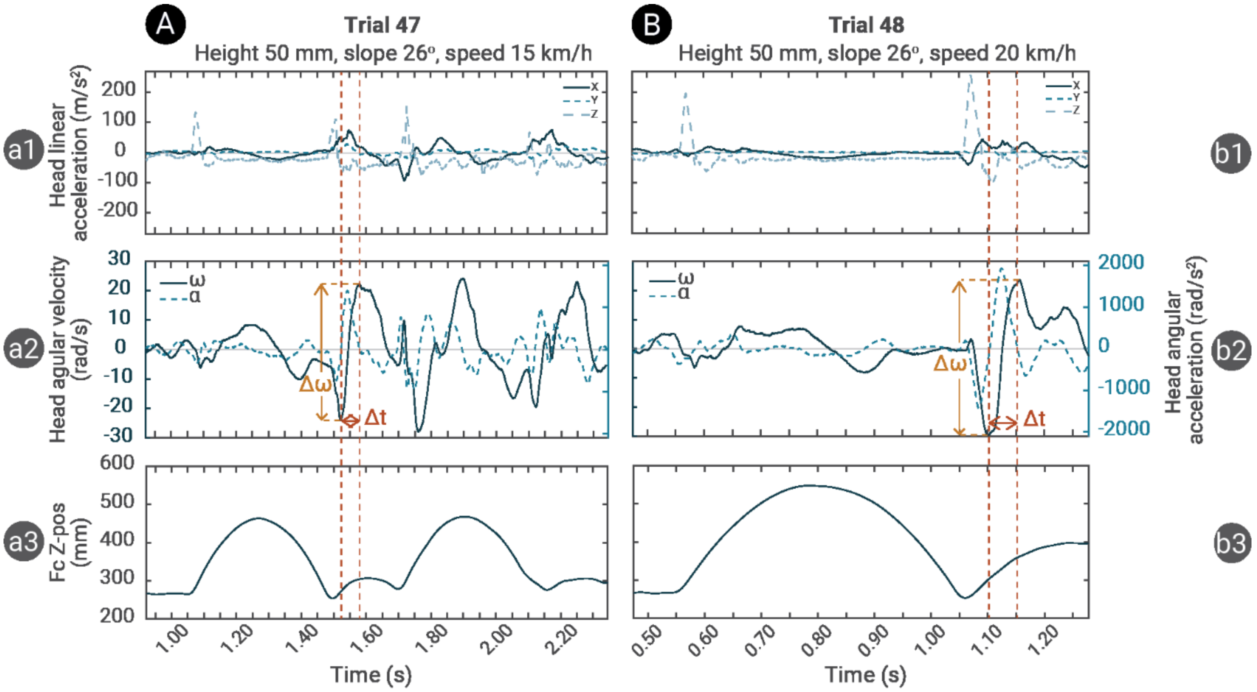

Figure I2 – Two typical example datasets of trials 47 (A) and 48 (B), with the same speedbump type (C: height 50 mm and slope 26°) but different trailer speeds (10 and 20 km/h, respectively). The local head linear xyz-accelerations (a1, b1), local head angular velocity and acceleration (a2, b2), and global Z-position of marker Fc (a3, b3) are all plotted against time in seconds. Vertical dashed red lines indicate the duration of the peak angular acceleration during the first bounce, Δt, and horizontal dashed orange lines indicate the change in angular velocity during that peak angular acceleration, Δω. The peak angular acceleration in trial 47 is approximately 1400 rad/s2 , with Δt approximately 50 ms, and Δω approximately 50 rad/s. The peak angular acceleration in trial 48 is approximately 1900 rad/s2 , with Δt approximately 50 ms, and Δω approximately 55 rad/s.

Typical ICOR

The ICOR is plotted with respect to the head's local reference frame for two typical trials in Figure I3.

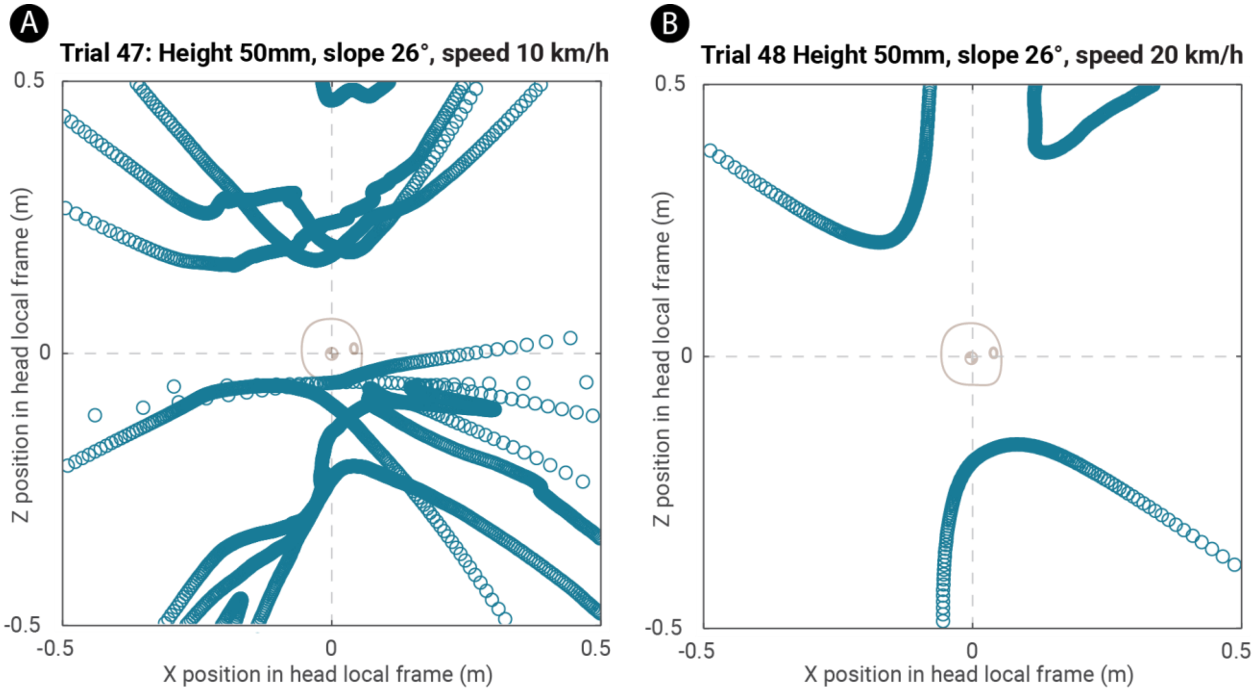

Figure I3 – Two scatter plots show typical examples of all the positions of the ICOR with respect to the CoM of the head during the complete trial, from the start of the scene until the end of the scene at 4.5 m. A: Trial 47, bump type C, trailer speed 10 km/h. B: Trial 48, bump type C, trailer speed 20 km/h. The icon of the dummy head is to scale with respect to the figure axes.

Trailer roll

The data of two typical trials where roll occurred is plotted in Figure I4A,B. Here, linear y-accelerations were observed in the local reference frames of the head and torso during landing.

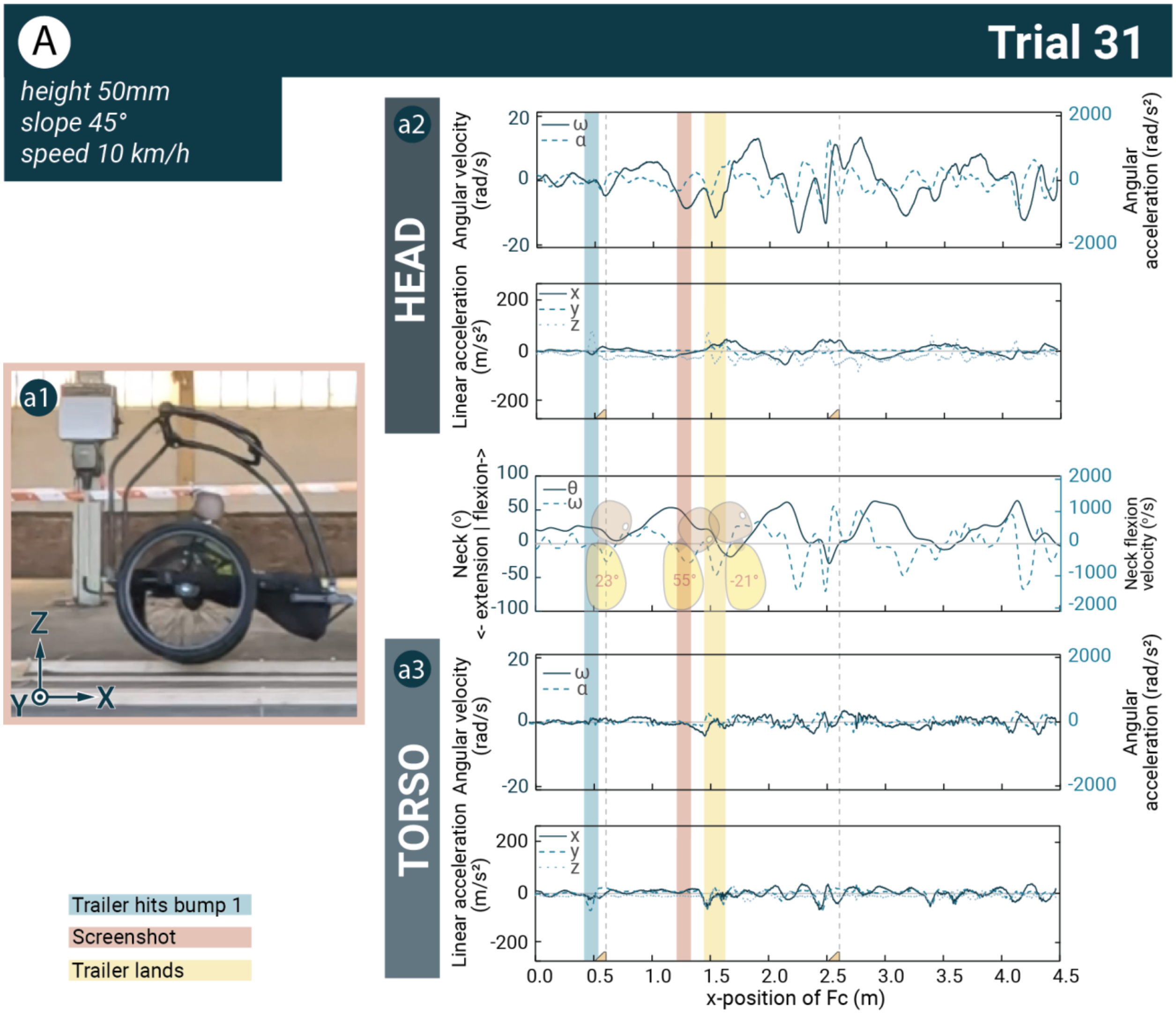

Figure I4A – Examples of trailer roll occurring during test conditions with bump type B (A) (height 50 mm, slope 45°, trial 31) and bump type E (B) (height 100 mm, slope 45°, trial 94), both at a speed of 10 km/h. (a1) and (b1) show a screenshot from video footage filmed with a GoPro camera, mirrored vertically to match the positive X-direction of the plots. (a2) and (b2) show plots of the head the head angular velocity and acceleration and linear acceleration. (a3) and (b3) show plots of the torso the head angular velocity and acceleration and linear acceleration. The wide light red vertical strips spanning (a2)-(a3) and (b2)-(b3) indicate the approximate timestamp that the corresponding screenshots in (a1) and (b1) were taken, respectively. Bump locations are indicated by the triangle shaped cross sections in (a3) and (b3) with a vertically ascending dashed line to indicate the back of the bumps.

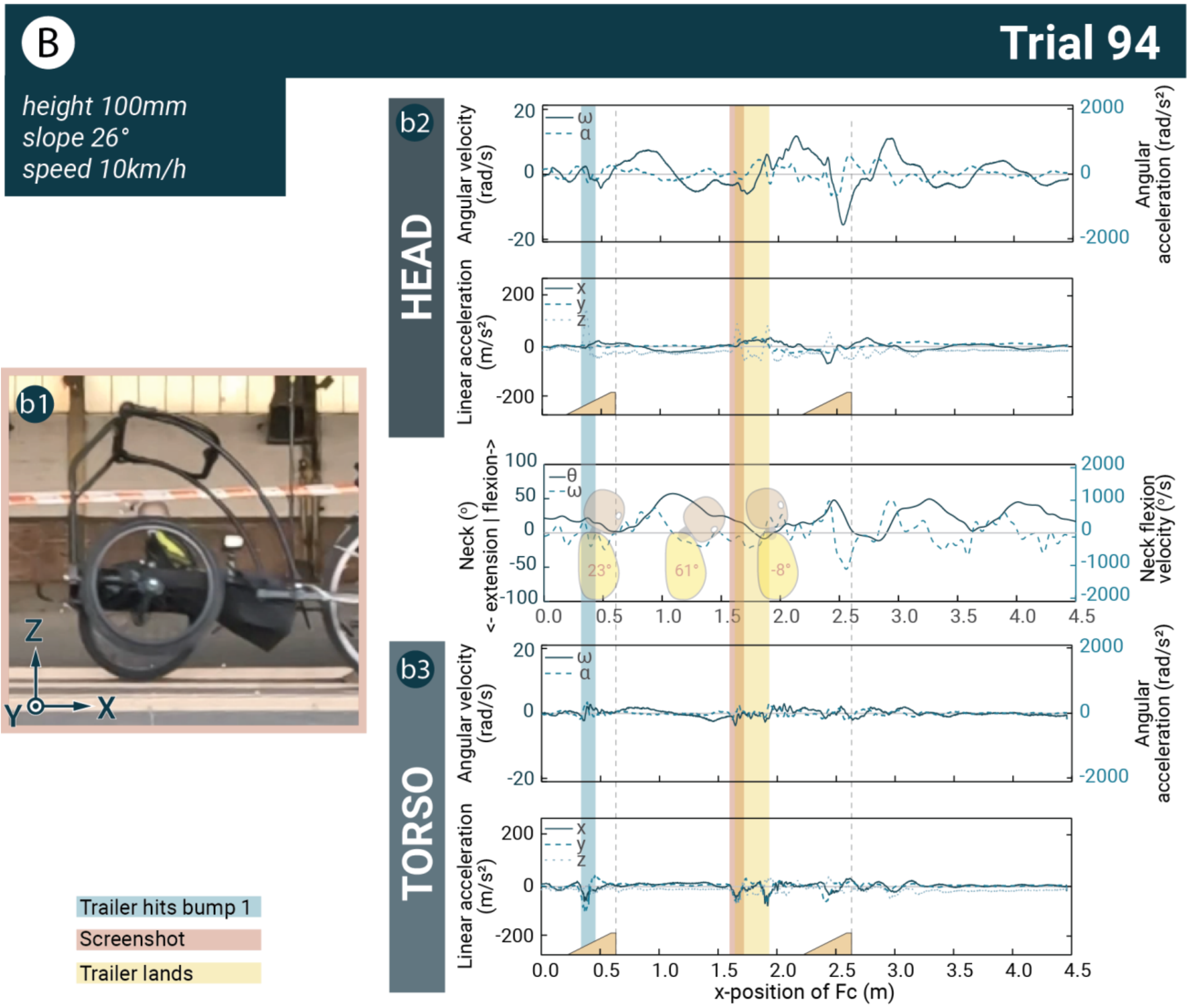

Figure I4B – See caption Figure I4A

Tire deformation

Screenshots from two trials of the same speed and with the same bump height, but with different bump slopes, are shown in Figure I5. These screenshots suggest that more local tire deformation occurred with the steeper bump slopes.

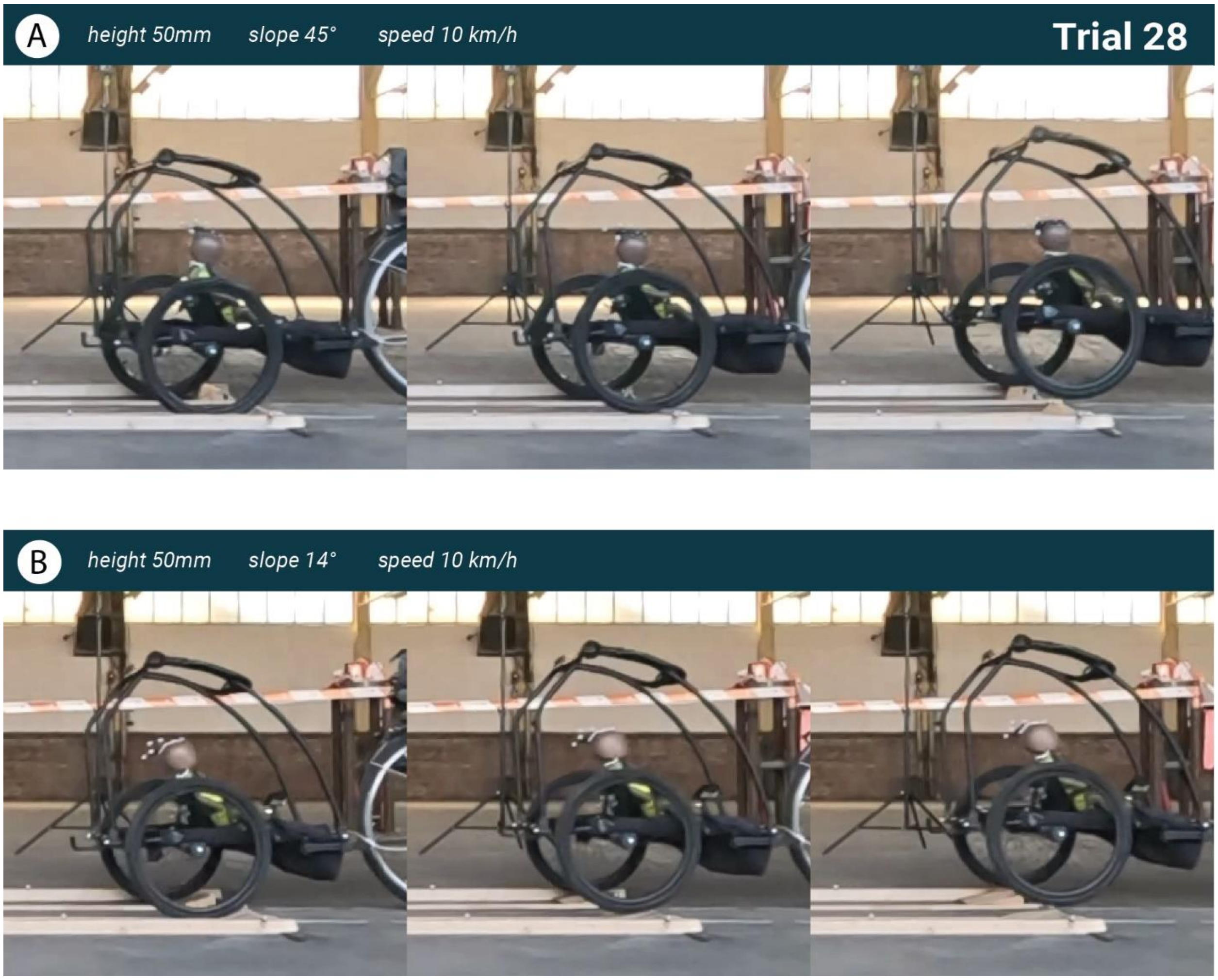

Figure I5 –The bicycle trailer hitting a bump with small (A) and large (B) slope angle, shown in three consecutive frames from the video taken from the side off the track (a1,a2,a3 and b1,b2,b3). Note the local deformation of the tire where it hits the steeper bump (a2) compared to the less steep bump (b2).
